# Plasmonic Au@Ag@mSiO_2_ Nanorattles for In Situ Imaging of Bacterial Metabolism by Surface-Enhanced Raman Scattering Spectroscopy

**DOI:** 10.1021/acsami.1c21812

**Published:** 2021-12-20

**Authors:** Sarah De Marchi, Daniel García-Lojo, Gustavo Bodelón, Jorge Pérez-Juste, Isabel Pastoriza-Santos

**Affiliations:** † CINBIO, Universidade de Vigo, Departamento de Química Física, Campus Universitario As Lagoas, Marcosende, 36310 Vigo, Spain; ‡ Galicia Sur Health Research Institute (IIS Galicia Sur), SERGAS-UVIGO, 36310 Vigo, Spain

**Keywords:** plasmonic silica nanorattles, pH monitoring, microbial colonies, SERS sensor, plasmonic hybrids

## Abstract

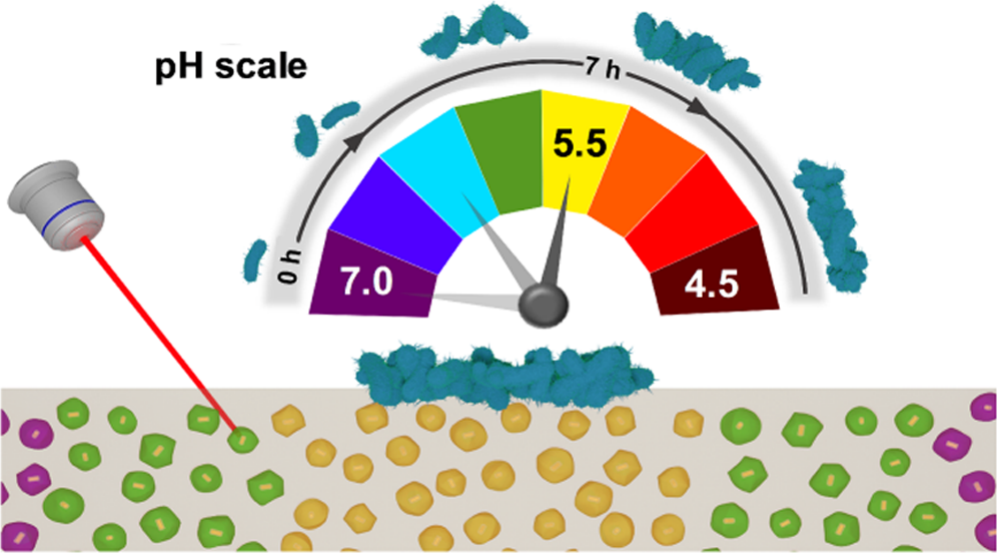

It is well known that microbial populations and their interactions are largely influenced by their secreted metabolites. Noninvasive and spatiotemporal monitoring and imaging of such extracellular metabolic byproducts can be correlated with biological phenotypes of interest and provide new insights into the structure and development of microbial communities. Herein, we report a surface-enhanced Raman scattering (SERS) hybrid substrate consisting of plasmonic Au@Ag@mSiO_2_ nanorattles for optophysiological monitoring of extracellular metabolism in microbial populations. A key element of the SERS substrate is the mesoporous silica shell encapsulating single plasmonic nanoparticles, which furnishes colloidal stability and molecular sieving capabilities to the engineered nanostructures, thereby realizing robust, sensitive, and reliable measurements. The reported SERS-based approach may be used as a powerful tool for deciphering the role of extracellular metabolites and physicochemical factors in microbial community dynamics and interactions.

## Introduction

Microbial biofilms, the most common form of existence of microorganisms in nature, are indispensable living entities governing the global biogeochemical cycle and the healthy activity of the microbiota.^1^ Biofilms can act as infectious agents,^2^ and they are responsible for biofouling and biocorrosion of materials, thereby causing a substantial economic burden in the industry.^3^ These microbial communities possess genotypic and physiologic traits different from their planktonic (e.g., free-living) counterparts being significantly more resistant to antibiotics and other chemical agents.^4^ Recently, the World Health Organization outlined antimicrobial resistance as one of the 10 major threats to global health.^5^ Hence, the study of microbial biofilms and their development is essential to understand this microbial way of life and thus facilitate strategies that allow their control and eradication.

Biofilms composed of multiple microbial species or by a single species are enclosed at high cell densities within a self-produced extracellular matrix. In such densely populated environments, as a result of their metabolic activities, microbes excrete bioactive chemical compounds that can act as cues and signals for intercellular communication,^6,7^ as well as metabolic byproducts that can also greatly influence the development and composition of biofilms.^8,9^ For instance, microbial fermentation can lead to the production of acids that can lower the local pH significantly. Such acidification can affect the physiological state of resident microbes,^10^ promote resistance to antibiotics,^11^ or induce enamel demineralization and dental caries.^12^ Notably, the detection of pH has been used as an indicator to reveal microbial growth. Moreover, biofilms are known to affect wound healing. The capacity to eradicate the microbes from the wound is significantly affected by changes in pH, as these will influence the antimicrobial efficacy of antibiotics.^13^ Thus, noninvasive and simultaneous monitoring of extracellular bioactive metabolites and physicochemical factors (e.g., pH) with spatial and temporal resolution can provide valuable information regarding the mechanisms that regulate the biogenesis, composition, and function of microbial communities.

The rapid development of nanotechnology and materials science in recent years has made possible the rational design and fabrication of a wide variety of nanostructured sensors for the noninvasive assessment of the cell. Compared with molecular probes, nanosensors can effectively enhance sensitivity, specificity, and targeting ability, as well as provide additional multimodal, multiplexing, and multifunctional capabilities.^14,15^

Several technologies have been reported for pH biosensing, including microelectrodes, nuclear magnetic resonance imaging, and field-effect transistors; however, these methods are limited by slow response times and low spatial resolution.^16^ Optical sensing of pH based on distinct absorption or fluorescence changes of reporter molecules upon their protonation/deprotonation at different pH values is gaining increasing attention owing to their noninvasive nature, high sensitivity, and spatiotemporal resolution.^17−23^ In this context, fluorescence microscopy is a popular technique that has been widely used to study the role of hydrogen ions and pH in physiological and pathological processes. Indeed, a wide array of fluorescent molecules, as well as fluorescent nanoparticle-based nanosensors, have been developed and successfully applied for real-time imaging of pH in biological systems.^24−26^

Surface-enhanced Raman scattering (SERS) spectroscopy excels for its ability to combine high sensitivity with rich vibrational information, enabling detection limits down to the single-molecule level under optimal conditions. The Raman signal can be excited with a wide range of wavelengths, shows higher photostability, and displays narrower bandwidth, which allows for simultaneous sensing of multiple analytes.^27^ This makes SERS a powerful technique for multiplex (bio)chemical analysis.^28^ However, in situ SERS detection of metabolic compounds is challenged by the biological matrix, which can hamper the interaction of the target analyte with the metal surface, as well as increase the background signal.^29,30^

The combination of plasmonic nanostructures with molecular sieve materials is a way to overcome the aforementioned limitations of SERS for bioanalysis. The porous size of certain materials, such as mesoporous silica (mSiO_2_), zeolites, metal-organic frameworks (MOFs), or covalent organic frameworks (COFs), among others, is similar to that of bioactive metabolites. Therefore, those materials could be applied as molecular sieves by size exclusion for SERS analysis when combined with Au or Ag nanostructures.^31^ These mesoporous structures allow diffusing only small molecules toward the plasmonic nanostructure, while keeping large biomolecules (peptides, proteins, etc.) away, thereby avoiding the need for sample pretreatment. Several reports have already shown the molecular sieving effect of mSiO_2_ and mesoporous TiO_2_ (mTiO_2_) in the SERS analysis of samples in complex media.^32−34^ For instance, core–shell Ag@mSiO_2_ nanoparticles have demonstrated good performance for selective sensing of organophosphorus pesticides in different complex vegetable matrices.^33^ In this context, plasmonic nanorattle structures with a characteristic core@void@shell (yolk–shell) configuration, are widely used materials in different fields due to the tailorability and functionality of both the core and hollow shells.^35−39^ In the particular case of SERS analysis, the mesoporous shell in a yolk–shell structure provides spatial confinement to the plasmonic core keeping colloidal stability while avoiding undesired interaction of biomolecules with the metal surface that can impair the reliability of the sensor.

Herein, we report on the synthesis of mSiO_2_ nanorattles containing single plasmonic core–shell Au@Ag nanoparticles (Au@Ag@mSiO_2_) as nanoprobes for the noninvasive detection of extracellular metabolism in bacterial cultures by SERS. Initially, we show the in situ detection of the secretion of pyocyanin metabolite in liquid cultures of *Pseudomonas aeruginosa* with a high dynamic range. We also demonstrate the application of plasmonic nanorattles encoded with a pH-dependent Raman active molecule, 4-mercaptobenzoic acid (4-MBA),^40^ and embedded in a block of nutrient agar as a multifunctional SERS platform for highly sensitive detection and spatiotemporal imaging of metabolic pH changes in colonies of *Escherichia coli*. Our results highlight the great potential of the Au@Ag@mSiO_2_ nanoparticles as a SERS sensor for diagnostic and environmental applications.

## Results and Discussion

**Figure 1:**
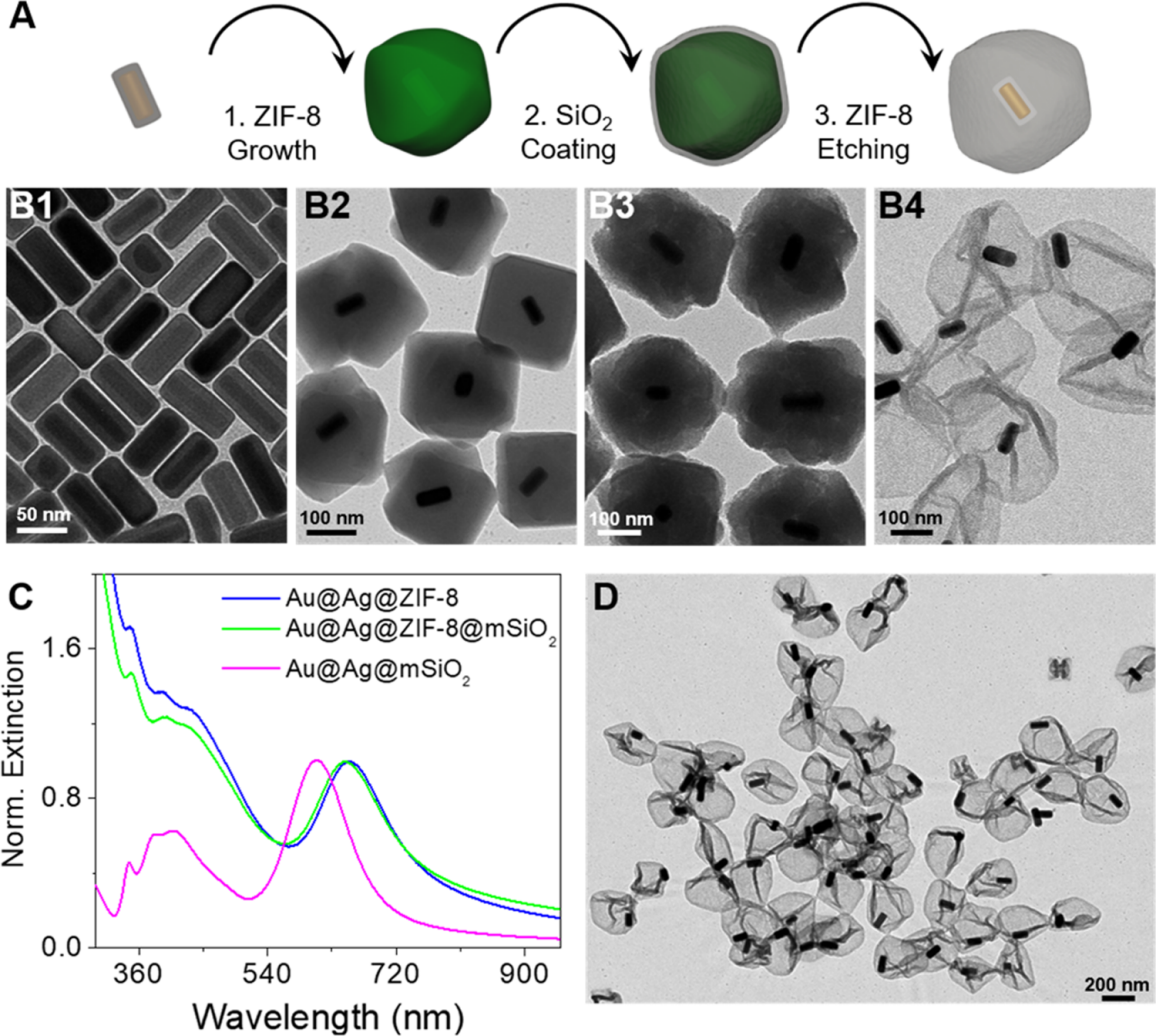
(A) Schematic representation of the multistep fabrication process of plasmonic mSiO_2_ nanorattles: ZIF-8 coating of Au@Ag nanorods, mSiO_2_ coating of Au@Ag@ZIF-8 nanoparticles, and ZIF-8 etching of Au@Ag@ZIF-8@mSiO_2_. (B) TEM images of Au@Ag nanorods (B1), Au@Ag@ZIF-8 nanoparticles (B2), Au@Ag@ZIF-8@mSiO_2_ nanoparticles (B3), and plasmonic mSiO_2_ nanorattles (B4). (C) Normalized extinction spectra of Au@Ag@ZIF-8 nanoparticles (blue), Au@Ag@ZIF-8@mSiO_2_ nanoparticles (green), and plasmonic mSiO_2_ nanorattles (pink). (D) Representative TEM image of plasmonic mSiO_2_ nanorattles.

Plasmonic Au@Ag@mSiO_2_ nanorattles were obtained via a multistep process where Au@Ag@ZIF-8 core–shell–shell nanocrystals acted as sacrificial templates (Figure 1A). The first step involves the single encapsulation of Au@Ag core–shell nanorods (Figure 1B1) within ZIF-8 nanocrystals (Figures 1B2 and S1A in the Supporting Information), as previously reported.^41^ Subsequently, the Au@Ag@ZIF-8 nanocrystals are coated with mSiO_2_ (with a pore size ∼3 to 4 nm)^42^ through a sol–gel process in the presence of cetyltrimethylammonium bromide (CTAB) (Figures 1B3 and S1B in the Supporting Information). As expected the silica coating led to a change in the ζ-potential from +31.0 ± 1.8 to −10.5 ± 0.4 mV. Finally, the plasmonic mSiO_**2**_ nanorattles (Au@Ag@mSiO_2_) were obtained through the selective etching of the ZIF-8 shell by acid treatment through the protonation of imidazolate ligands from the MOF matrix. After ZIF-8 etching, a further decrease of the ζ-potential was observed (−18.4 ± 0.8 mV), probably as a consequence of CTAB removal. The process was also studied by ultraviolet–visible–near infrared (UV–vis–NIR) spectroscopy. As shown in Figure 1C, while the mSiO_2_ coating leads to a slight blueshift in the localized surface plasmon resonance (LSPR) of Au@Ag@ZIF-8 nanocrystals due to scattering effects, the etching of the ZIF-8 shell strongly affects the optical response of the nanocrystals. Thus, the main LSPR band is blue-shifted from 648 to 607 nm, produced by the lower refractive index of the solvent (water, 1.333) compared with ZIF-8 (∼1.54), and the extinction at short wavelengths decreases due to the diminution of scattering effects. Additionally, the dissolution of the ZIF-8 shell and the formation of plasmonic mSiO_**2**_ nanorattles were confirmed by transmission electron microscopy (TEM) analysis (Figures 1B4,D and S1C in the Supporting Information). The majority of the nanorattles are formed by a silica capsule containing one Au@Ag nanoparticle in their interior. Moreover, the analysis reveals the presence of folds and creases resulting from the collapse of mSiO_**2**_ nanorattles after their air-drying. These results were further confirmed by energy-dispersive X-ray (EDX) elemental analysis (see Figure S2 in the Supporting Information).

**Figure 2:**
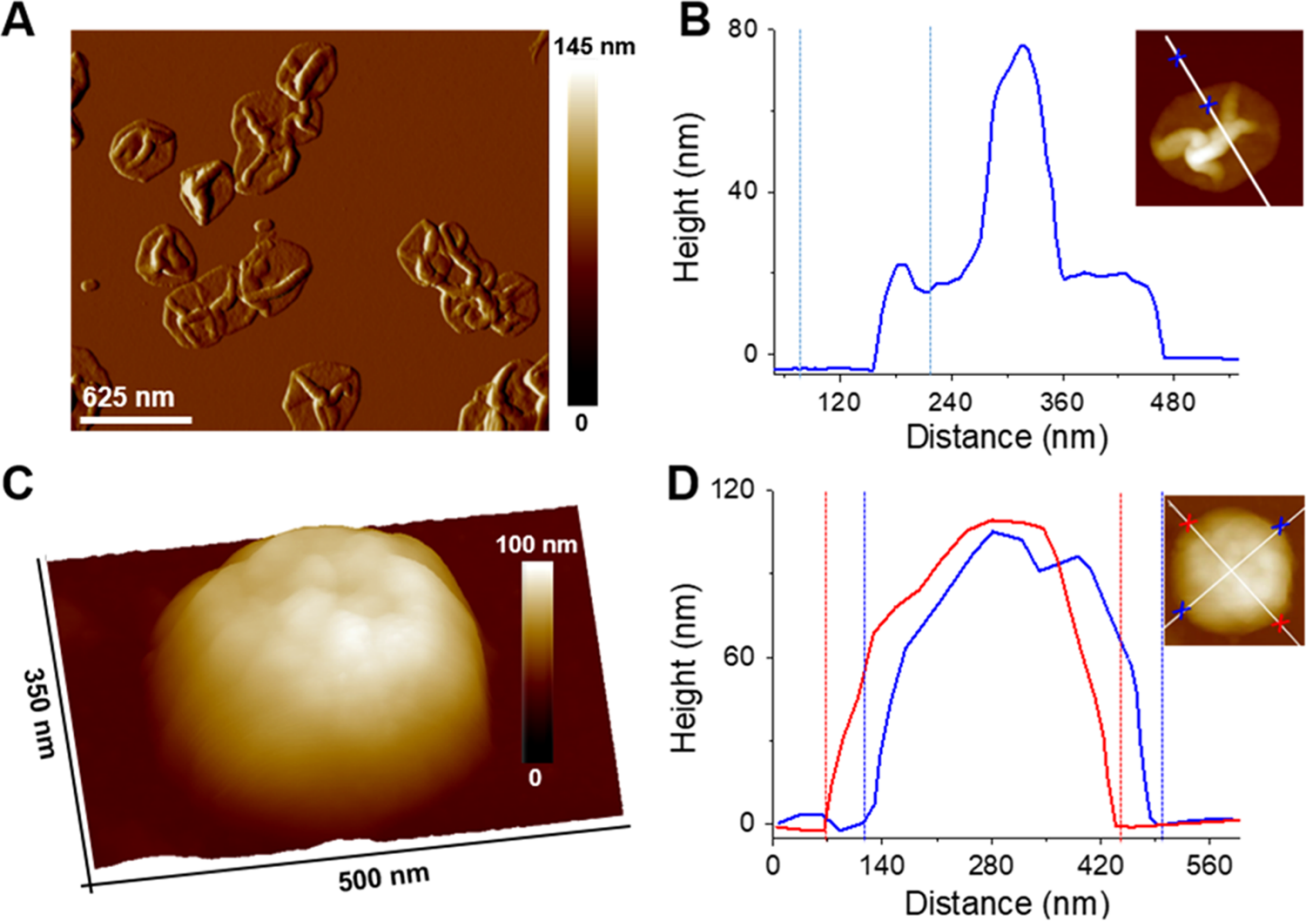
(A) AFM topographic amplitude image of multiple plasmonic mSiO_2_ nanorattles in the dried state. (B) Height profile of a plasmonic mSiO_2_ nanorattle in the dried state. (C, D) Representative AFM topographic three-dimensional (3D) height image (C) and height profile (D) of a hydrated plasmonic mSiO_2_ nanorattle.

Atomic force microscopy (AFM) was used to estimate the wall thickness of the mesoporous silica shell and the dimensions of the nanorattles (Figures 2 and S3 and S4 in the Supporting Information). The analysis of AFM height profiles performed on the dried nanorattles (Figures 2A,B and S3 in the Supporting Information) indicates an average mSiO_2_ shell thickness of 10.1 ± 1.1 nm, determined as half of the height in the collapsed flat region. On the other hand, the analysis of hydrated nanorattles shows an average diameter of 342.3 ± 38.3 nm, estimated as the horizontal distance measured in the height profile (Figures 2C,D and S4 in the Supporting Information). This value is in agreement with the dimensions of the Au@Ag@ZIF-8 obtained by TEM analysis. It should be noted that the clear differences between the dried and hydrated states demonstrate the flexibility of the mesoporous silica capsules.

**Figure 3:**
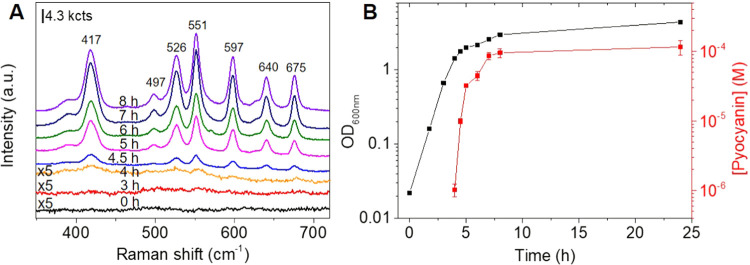
(A) SERS analysis of pyocyanin secreted by the *P. aeruginosa* PA14 strain at different growth times. For clarity, the spectra noted with ×5 were multiplied by a factor of 5. (B) Growth curve of *P. aeruginosa* PA14 by measuring the optical density of the liquid culture at 600 nm (black squares) and amount of pyocyanin excreted by *P. aeruginosa* PA14 determined by SERS as a function of time (red squares). The error bars represent the standard deviation of three different measurements. All SERS measurements were carried out with an excitation laser line at 785 nm employing a 15× objective, maximum power of 53.1 mW, and an acquisition time of 10 s.

Next, we evaluated the capability of the plasmonic mSiO_2_ nanorattles for SERS detection in a complex biological medium. Selecting as model metabolite commercial pyocyanin, we studied first the sensing capabilities of the plasmonic nanorattles in water as well as in lysogeny broth (LB), a complex nutrient medium commonly used for culturing bacteria. The SERS spectrum of pyocyanin shows a group of peaks between 400–600 cm^–1^ corresponding to different ring deformations (Figure S5A in the Supporting Information).^34^ The SERS data analysis of samples containing different concentrations of pyocyanin shows that the metabolite in water can be quantitatively detected in a concentration range from 20 nM to 10 μM (Figure S5A,B in the Supporting Information), while in LB medium it is detected in a concentration range from 0.5 to 50 μM, fitting in both cases extremely well to a Langmuir isotherm (Figure S5C,D in the Supporting Information). Subsequently, the detection of pyocyanin excreted by *P. aeruginosa* bacteria to the culture medium was investigated. To this aim, sample aliquots were collected at different time intervals of bacteria growth, mixed with the plasmonic mSiO_2_ nanorattles, and analyzed by SERS and UV–vis-NIR spectroscopy without any sample treatment. Figure 3A shows the time evolution of the SERS spectra during the bacteria growth where the presence of pyocyanin is evidenced after 4 h, corresponding to 1.15 × 10^9^ CFU/mL (estimated from the value of the optical density at 600 nm, OD_600nm_, 1.434, (Figure 3B)). No evidence of signals from other molecules secreted by bacteria are observed most probably due to the sieving effect of the mSiO_2_ (see Figure S6 in the Supporting Information).^32^ The interpolation of the SERS intensity data at 675 cm^–1^ to the calibration curve obtained with commercial pyocyanin in LB medium (Figure S5D in the Supporting Information) allows obtaining the evolution curve of the production of pyocyanin. As shown in Figure 3B, at 4 h the concentration of pyocyanin secreted by *P. aeruginosa* was approximately 1 μM, and subsequently, it increased abruptly during the exponential growth of *P. aeruginosa*. Notably, no pyocyanin could be detected at 4 h of bacterial growth by UV–vis-NIR spectroscopy (Figure S7 in the Supporting Information), which indicates the higher sensitivity of our SERS sensor and its suitability to detect pyocyanin at the early stages of bacterial growth. As expected, control experiments performed with surfactant-stabilized Au@Ag nanorods show their limitation to detect pyocyanin in LB medium due to the lack of colloidal stability (Figure S8 in the Supporting Information).

Once we confirmed the suitability of colloidal Au@Ag@mSiO_2_ nanorattles toward plasmonic detection of a bacterial metabolite in growth medium, we explored their application for monitoring extracellular pH changes that result from the metabolic activities of bacteria. The fermentation of glucose by *E. coli* leads to the production of lactic and acetic acid that can contribute to the acidification of the external medium.^43^ For obtaining a SERS-active pH nanosensor, the plasmonic surface of the mSiO_2_ nanorattles was functionalized with a pH-sensitive molecular probe such as 4-MBA. Since its SERS features strongly depend on the pH of the surrounding environment,^44^ 4-MBA has been used to fabricate SERS-based pH nanosensors.^45−48^ The 4-MBA encoding was studied by SERS (Figure S9A,B in the Supporting Information) suggesting the rapid diffusion of this molecule through the mesoporous SiO_2_ shell and its attachment onto the metallic surface to form a monolayer within the first 10 min. Typical SERS spectra of 4-MBA recorded in phosphate buffer at different pHs are shown in Figure S9C in the Supporting Information. The peaks at 695, 848, 1430, and 1710 cm^–1^ are assigned to the vibrational modes of the pH-sensitive carboxylic moiety of 4-MBA. While the bands at 848 cm^–1^ (COO^–^ bending) and 1430 cm^–1^ (COO^–^ stretching) arise in an alkaline environment when the molecule is deprotonated, those at 695 cm^–1^ (COOH stretching) and 1710 cm^–1^ (C=O stretching) appeared when the molecule is protonated in an acid environment.^44^ Note that the two prominent spectral bands at 1076 and 1586 cm^–1^, attributed to the aromatic ring breathing mode, are not pH-sensitive.^47^ In the present study, we focused on the 695 and 848 cm^–1^ spectral bands. To evaluate the performance of the plasmonic mSiO_2_ nanorattles as a pH sensor, they were suspended in a series of buffered solutions with verified pHs ranging between 2.5 and 12 (see the Experimental Section) and we monitored the variation of the relative ratio of the areas of the bands at 695 (A_695_) and 848 cm^–1^ (A_848_) as described elsewhere^44^
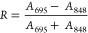
1

By plotting *R* as a function of pH (see Figure S9D in the Supporting Information), we obtained a calibration curve that follows the typical trend of the Henderson-Hasselbalch equation and that fitted to a sigmoidal equation revealing a linear region in the pH range from 5.0 to 8.0. In addition, compared with typical core–shell Au@Ag@mSiO_2_ nanoparticles, the plasmonic Au@Ag@mSiO_2_ nanorattles featured higher sensitivity (see further details in Figure S10 in the Supporting Information).

Next, the MBA-encoded plasmonic nanorattles were tested for monitoring the changes in the extracellular pH generated as a result of bacterial metabolism in a liquid medium. The pH nanosensors were incubated with aliquots taken from cultures of planktonic *E. coli* grown overnight in LB supplemented, or not, with 2% of glucose (Figure S11A in the Supporting Information). As shown in Figure S11A in the Supporting Information, the fermentation of glucose by *E. coli* induced acidification of the medium protonating the carboxylic group of the Raman reporter detected by the appearance of the peak at 695 cm^–1^ (COOH stretching) and the disappearance of the peak at 848 cm^–1^ (COO^–^ bending). These spectral changes were not observed in the bacterial culture grown in LB without glucose, used as a control. Interpolation of the SERS data to the calibration curve (Figure S9D in the Supporting Information) yielded pH values of ca. 5.0 and 7.3 in the culture media with and without glucose, respectively. These values of pH were further confirmed using a conventional pH meter, thereby demonstrating the reliability of the nanosensor. To confirm that the 4-MBA spectral changes were produced by the metabolic activities of bacteria, further experiments were carried out with *P. aeruginosa* (Figure S11B in the Supporting Information), a nonfermenter microbe unable to lead to an acidification of the medium in the presence of glucose. As expected, no significant spectral variations were observed in the presence of glucose.

**Figure 4:**
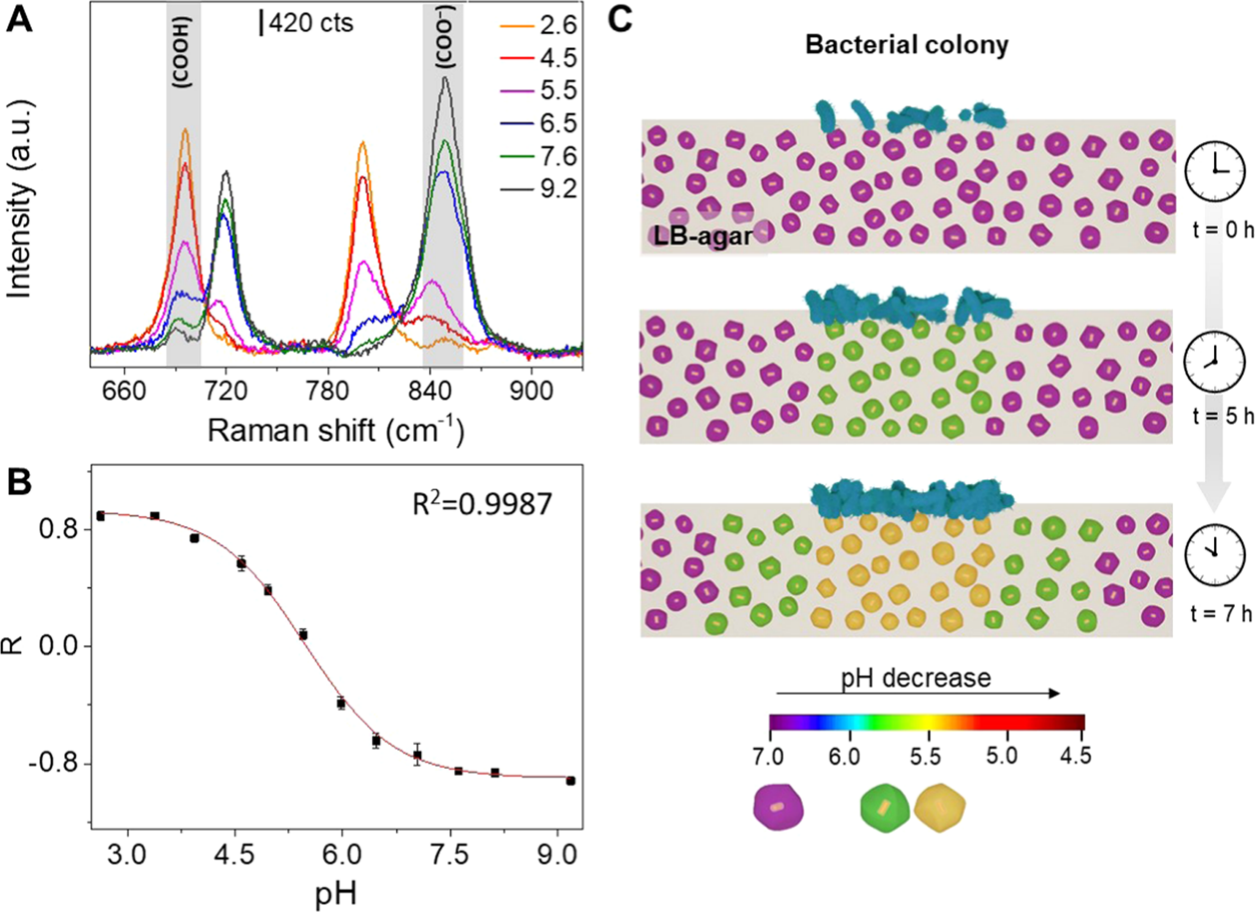
(A) Representative SERS spectra of nanoratles@LB-agar substrates at different pHs. (B) Experimental calibration curve of ratio *R* = (A_695_ – A_848_)/(A_695_ + A_848_) as a function of pH. The error bars show the standard deviation from the mean of *n* = 15 measurements. SERS measurements were carried out at 785 nm with a 10× objective, 8.22 mW maximum power, 3 accumulations, and an acquisition time of 10 s. (C) Schematic illustration of in situ SERS monitoring of pH changes as a result of bacterial metabolism.

In nature, bacteria often live as densely packed colonies subjected to multiple growth constraints that are absent in planktonic cultures.^49^ In solid media, nutrients must diffuse through the matrix into the colonies, whereas metabolic end-products diffuse away.^50^ In particular, the outward diffusion of metabolic acids generates pH gradients that influence the development and composition of the microbial populations.^10,51^ Intending to monitor the extracellular pH of bacterial colonies, we fabricated a hybrid substrate that consists of 4-MBA-encoded plasmonic nanorattles embedded in an agar matrix containing nutrients (i.e., LB). Agar is a gelling agent commonly used as a support matrix for growing bacteria in vitro. Its macroporous structure and its large water fraction facilitate nutrient uptake, as well as diffusion of metabolites and other chemical species to the local environment. The 4-MBA-encoded plasmonic nanorattles were embedded by simple addition to molten LB-agar. As shown in Figure S12A in the Supporting Information, nanoparticles are homogeneously distributed in the agar layer. The substrate, termed nanorattles@LB-agar, displays an optical response similar to the colloidal nanorattles in water with the main LSPR band centered at 608 nm, but with an increase of extinction especially at shorter wavelengths attributed to the scattering of light by the LB-agar matrix (Figure S12B in the Supporting Information). The pH sensing capabilities of the nanorattles@LB-agar substrates were tested by adjusting the pH of the molten LB-agar medium in the range of 2.6–9.2 (see Experimental Section for further details). Like the colloidal nanorattles, the nanorattles@LB-agar can efficiently produce pH-sensitive signals over a physiologically relevant range (Figure 4A). Remarkably, the calibration curve obtained (Figure 4B) demonstrates the high performance of this substrate. Besides, further experiments performed to test the repeatability and uniformity of the substrates (Figure S13 in the Supporting Information) confirm their potential as robust and reliable pH sensors.

**Figure 5:**
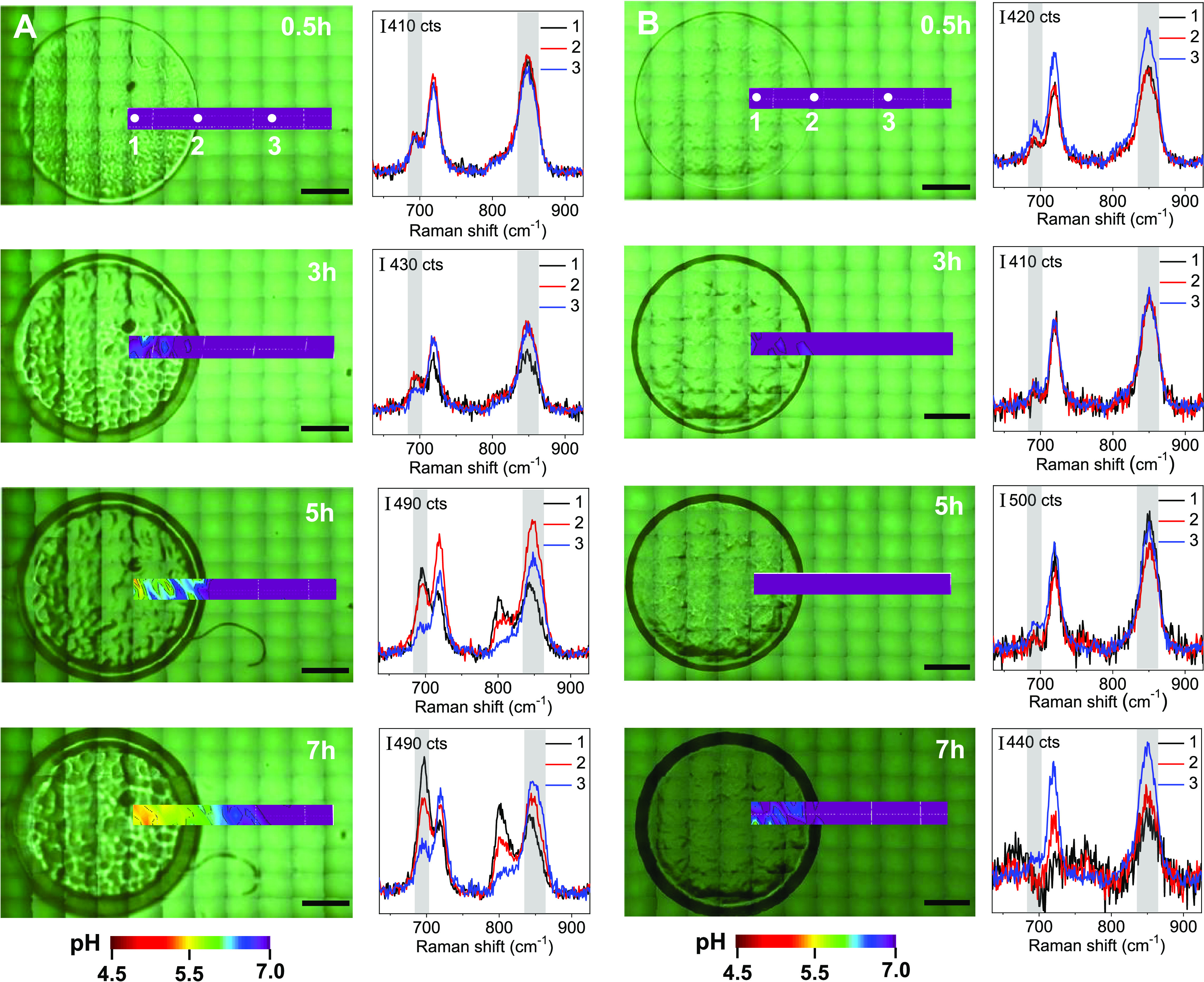
Spatiotemporal pH distribution maps of a selected area of the nanorattles@LB-agar substrate supplemented with glucose (A) and without glucose (B) during the growth of a colony of *E. coli*. SERS mappings were measured at 0.5, 3, 5, and 7 h, as indicated. Representative SERS spectra were recorded at the points 1, 2, and 3 (indicated by white dots) of maps shown in (A) and (B). The grey shaded regions indicate the pH-sensitive bands at 695 and 848 cm^–1^ taken to estimate the pH through interpolation of the relative ratio of their areas (A_695_ and A_848_) in the calibration curve. Scale bars correspond to 1 mm. SERS measurements were carried out at 785 nm with a 10× objective, 8.22 mW maximum power, 10 accumulations, and acquisition time of 1 s.

Since the nanorattles@LB-agar substrates can support bacterial growth as colonies (Figure S14 in the Supporting Information), we next assessed the suitability of the plasmonic platform for space- and time-resolved monitoring of microbial metabolism in situ by SERS during the growth of *E. coli* bacteria, as a single colony, with and without glucose (Figures 4C and 5). As shown in the scheme of Figure 4C, as the population of bacteria increases with time, a gradient of pH should appear as a consequence of glucose fermentation and the diffusion of metabolic acids. To prove that, we performed SERS mappings over a selected area in the plasmonic platform for 7 h (Figure 5A) and then the pH was estimated by interpolating the relative ratio of the areas of the bands at 695 (A_695_) and 848 cm^–1^ (A_848_) in the calibration curve from Figure 4B. Figure 5A shows the time evolution of the extracellular pH distribution mappings monitored in a selected area of the nanorattles@LB-agar substrate during the growth of an individual colony of *E. coli*. Figure 5A also shows representative SERS spectra recorded at different growth times of the *E. coli* colony in three different points of the nanorattles@LB-agar substrate; (1) inside the colony, (2) at the border of the colony, and (3) a few millimeters far from the colony, as indicated. The pH-sensitive bands at 695 and 848 cm^–1^ were taken to estimate the pH. While at 0.5 h of growth the data analysis reveals a homogeneous pH value of 7.0 (Figure 5A), the time-course SERS analysis demonstrates the progressive acidification of the extracellular medium and a pH gradient within the colony toward its margins. After 7 h of growth, it was detected a substantial decrease in the local pH, which achieved values as low as 5.5 in the interior of the colony. As expected, the pH distribution mappings obtained in the absence of glucose did not show any significant change of the pH during the growth of a single *E. coli* colony (Figure 5B) in the recorded spectra throughout the growth time analyzed, demonstrating that the pH changes measured in our system were indeed produced by the fermentation of glucose. These results are further supported by additional experiments shown in the Supporting Information (Figures S15 and S16 in the Supporting Information). Importantly, we evaluated the signal stability of the nanorattles@LB-agar during bacterial growth by acquiring SERS spectra at 0.5, 3, 5, and 7 h, in distant regions from colonies, and using substrates without glucose. As shown in Figure S13C,D in the Supporting Information, no significant changes are observed in the SERS spectra or the corresponding ratio *R* = (A_695_ – A_848_)/(A_695_ + A_848_) which further support the reliability of these platforms for pH sensing. The utility of SERS for measuring pH has been demonstrated in mammalian cells cultured in vitro.^45−48,52−56^ However, to our knowledge, this is the first report describing the application of SERS for monitoring pH changes in bacterial colonies. Homeostasis of the bacterial pH is key for the regulation of important cellular processes including gene expression, enzymatic function, metabolism, motility, and division.^57^ To achieve pH homeostasis, bacterial cells possess regulatory networks that govern the expression of distinct sets of genes under acid and alkaline conditions. In this context, bacteria can metabolically adapt to sublethal environmental acid stress (pH 5.5–4.5) by inducing an adaptive tolerance response,^58^ which has been linked to antibiotic resistance.^59^ Moreover, acidic pH sensing is required for virulence.^60^ Also, the detection of pH changes as a result of microbial metabolism during growth is a means to assess the bacterial response to antibiotics.^61^ Traditionally, the molecular aspects of pH homeostasis in bacteria have been elucidated through genetic and molecular biology tools.^57^ However, these methods are generally destructive as they involve the preparation of cellular extracts for biochemical analysis. In this context, various fluorescence-based approaches have been developed to noninvasively monitor pH changes in bacterial populations.^62^ The aforementioned analytical approaches have limited multiplex capabilities and generally only allow monitoring a single parameter (e.g., pH). As shown herein, the plasmonic Au@Ag@mSiO_2_ nanorattles enabled not only the sensing of pH in bacterial colonies but also the detection of secreted metabolites (e.g., pyocyanin) in bacterial cultures, highlighting the promising potential of the nanosensor for multiplex sensing of bacterial metabolism by SERS.

## Conclusions

In conclusion, we developed a SERS-based approach for the space- and time-resolved in situ monitoring of microbial metabolism. The method relies on plasmonic Au@Ag@mSiO_2_ nanorattles comprising a single plasmonic nanoprobe enclosed within a mesoporous silica capsule that can efficiently enhance the Raman signal of pyocyanin and 4-MBA in a complex biological medium (i.e., LB) bearing high ionic strength. As reported in this work, the silica capsule is a key element that facilitates robust and reliable SERS measurements as it provides colloidal stability and molecular sieving capabilities. The intrinsic features of the engineered nanorattles enable their incorporation into an agar matrix for the fabrication of a biocompatible plasmonic substrate (i.e., nanorattles@LB-agar) that supports bacterial growth as colonies and cellular metabolism. We demonstrated that this hybrid plasmonic sensor allowed spatial biosensing of extracellular pH in colonies of *E. coli*, as well as sensing of secretion of pyocyanin. Importantly, SERS sensing of pH in bacterial colonies is shown for the first time. The reported SERS substrate shows not only a strong signal but also a very good linear response to pH within a wide range. Our approach based on plasmonic Au@Ag@mSiO_2_ nanorattles may also be adapted for real-time imaging of the biodistribution of other metabolites, volatile compounds, etc. that result from cellular metabolism. We envision that this new powerful tool will aid to gather new insights regarding the role of extracellular metabolism in microbial interactions and virulence, as well as in the development of antimicrobial therapies.

## Experimental Section

### Materials

Cetyltrimethylammonium bromide (CTAB, 98%), cetyltrimethylammonium chloride (CTAC, 25% wt solution), sodium borohydride (NaBH_4_, 99%), gold (III) chloride trihydrate (HAuCl_4_·3H_2_O, 99.9%), silver nitrate (AgNO_3_), l-(+)-ascorbic acid, 2-methylimidazole (2-MeIM, 99%), zinc nitrate hexahydrate (Zn(NO_3_)_2_·6H_2_O, 99%), methanol, ethanol, tetraethyl orthosilicate (TEOS), hydrochloric acid, agar, tryptone, yeast extract, sodium chloride, sodium dihydrogen phosphate, sodium phosphate dibasic, 4-mercaptobenzoic acid (4-MBA), and pyocyanin were purchased from Sigma-Aldrich. Milli-Q water was used in all experiments.

### Characterization

UV–vis–NIR absorption spectra were recorded using an Agilent 8453 spectrophotometer. Transmission electron microscopy was performed using a JEOL JEM 1010 microscope operating at an acceleration voltage of 100 kV. EDX analysis was performed using a JEOL 2100F field emission electron microscope equipped with an energy-dispersive X-ray (EDX) spectrometer, operating at an accelerating voltage of 200 kV. AFM images were collected on dry samples using a Multimode 8 Nanoscope V (Veeco) in the tapping mode and an NCHV-A cantilever (antimony (n)-doped Si, tip ROC < 10 nm, *K* = 40 N m^–1^, frequency 339–388 KHz). In the case of hydrated samples, AFM images on were recorded using the Peak Force QNM mode and a ScanAsyst-Fluid cantilever (silicon nitride, tip ROC < 10 nm, *K* = 0.7 N m^–1^, frequency 150 KHz).

### Synthesis of Au Nanorods

Au nanorods (Au NRs) were synthesized following a previously reported seed-mediated method.^63^ Gold seeds were prepared by fast reduction of HAuCl_4_ (10 mL, 0.5 mM) in 0.1 M CTAB aqueous solution upon addition of 460 μL of freshly prepared NaBH_4_ (0.01 M dissolved in 0.01 M NaOH) under vigorous stirring. The color of the solution changed from yellow to brownish-yellow and the seed solution was aged at 27 °C for 30 min before use. Separately, a growth solution was prepared by adding silver nitrate (70 μL, 0.1 M) to HAuCl_4_ solution (10 mL, 0.5 mM) in 0.1 M CTAB, followed by the addition of hydroquinone (500 μL, 0.1 M). The resulting mixture was hand-stirred until it became clear. Next, 160 μL of seed solution was added to the growth solution. The mixture was mixed thoroughly and left undisturbed overnight at 27 °C. The Au NRs were collected by centrifugation (8000 rpm, 20 min) and washed twice with 10 mL of CTAC (80 mM). Finally, the Au NRs were redispersed in 10 mL of CTAC (80 mM).

### Synthesis of Au@Ag Core–Shell Nanorods

Au@Ag core–shell nanorods were synthesized following a previously reported method with slight modifications.^64^ Briefly, 10 mL of the CTAC stabilized Au NRs was diluted to 40 mL of CTAC (80 mM) followed by the addition of 3.5 mL of ascorbic acid solution (0.1 M) and 3.5 mL of AgNO_3_ (0.01 M). The resulting solution was placed in an oven at 60 °C for 3 h. After cooling down to room temperature, the Au@Ag nanorods were washed twice with 10 mL of Milli-Q water (7000 rpm, 20 min) and finally redispersed in 10 mL of Milli-Q water. The final CTAC concentration was adjusted to 0.6 mM.

### Synthesis of Au@Ag@ZIF-8 Nanoparticles

Au@Ag@ZIF-8 nanoparticles were prepared as described elsewhere.^41^ In brief, 0.144 mL of CTAB (1 mM) was added to 1 mL of an aqueous solution of 2-methylimidazole (1.32 M) and stirred for 5 min. Then, 1 mL of Zn(NO_3_)_2_·6H_2_O (24 mM) and 1 mL of Au@Ag nanorods (final CTAC concentration, ca. 0.6 mM) were sequentially added to the mixture, stirred for 5 min, and left undisturbed for 3 h. The resulting Au@Ag@ZIF-8 nanoparticles were washed once with 10 mL of methanol (5500 rpm, 5 min) and finally redispersed in 3.14 mL of methanol.

### Synthesis of Au@Ag@mSiO_2_ Nanorattles

Au@Ag@mSiO_2_ nanorattles were prepared using a template selective etching approach. First, Au@Ag@ZIF-8 nanoparticles were coated with a thin layer of mesoporous silica following a previously reported protocol with modifications.^65^ In a typical experiment, 4 mL of Au@Ag@ZIF-8 nanoparticles was centrifuged at 5500 rpm for 5 min, dried at 60 °C for 1 h, and subsequently dispersed in 16 mL of a solution containing 1.5 mM CTAB and 8.25 mM 2-methylimidazole. Mesoporous silica coating was then carried out by adding three aliquots of TEOS (25 μL, 10 vol% in ethanol) at 60 min intervals to the colloidal suspension under stirring. After the third addition of TEOS, the mixture was stirred overnight. The resulting Au@Ag@ZIF-8@mSiO_2_ nanoparticles were washed twice with 10 mL of ethanol (6000 rpm, 10 min) and finally dispersed in 2 mL of ethanol. Next, the selective etching of ZIF-8 to obtain the Au@Ag@mSiO_2_ nanorattles was performed using HCl. Au@Ag@ZIF-8@mSiO_2_ nanoparticles (2 mL) were centrifuged (6000 rpm, 10 min), and the pellet was dispersed in 5 mL of Milli-Q water, followed by the addition of 3 mL of 0.06 M HCl. The suspension was sonicated for 15 min, and the final Au@Ag@mSiO_2_ nanorattles were washed three times by centrifugation (5000 rpm, 10 min) with 10 mL of ethanol and finally redispersed in 2 mL of ethanol.

### Synthesis of Core–Shell Au@Ag@mSiO_2_ Nanoparticles

In a typical experiment, Au@Ag nanoparticles (8 mL) dispersed in CTAC (0.6 mM) were centrifuged and redispersed in 25 mL of CTAB (6 mM). Then, 12 mL of ethanol, 200 μL of 2-methylimidazole, and 82 μL of TEOS (40% in ethanol) were sequentially added under stirring to the mixture. After stirring overnight, the particles were washed two times with ethanol and finally redispersed in ethanol.

### Calibration Curve for pH Monitoring Using Colloidal Au@Ag@mSiO_2_ Nanorattles

First, 10 sample aliquots of 100 μL Au@Ag@mSiO_2_ nanorattles were mixed with 100 μL of a 5 mM ethanolic solution of 4-MBA. After 45 min, each aliquot was centrifuged (4500 rpm, 5 min) twice, the first time the pellet was redispersed in 1 mL of water and the second time in 20 μL of water by sonication. Subsequently, the colloids were mixed with 500 μL of different 100 mM PB buffer solutions with pH values ranging from 2.5 to 12. Finally, all of the samples were analyzed by SERS.

### Calibration Curve for pH Monitoring in Au@Ag@mSiO_2_ Nanorattles@LB-Agar Substrates

First, 10 aliquots of 300 μL of Au@Ag@mSiO_2_ nanorattles were mixed with 300 μL of a 5 mM ethanolic solution of 4-MBA. After 45 min, each aliquot was centrifuged (4500 rpm, 5 min) twice, the first time the pellet was redispersed in 1 mL of water and the second time in 20 μL of water by sonication. Separately, a series of buffered lysogeny broth (LB) medium was prepared by dissolving tryptone (40 mg), yeast extract (20 mg), and sodium chloride (40 mg) in 4 mL of 100 mM PB solutions with pH values ranging from 2.6 to 9.2. The pH of the resulting solutions was confirmed using a pH meter. Next, 60 mg of agar were added and the mixture was heated to dissolve the agar. Immediately after, 100 μL of molten buffered LB-agar media was added to the MBA-encoded nanorattles (20 μL) and the mixture was transferred to a PDMS mold (1 × 1 × 0.5 cm^3^) placed over a glass slide. Once solidified, the different pH-adjusted nanorattles@LB-agar substrates were analyzed by SERS.

### Preparation of Au@Ag@mSiO_2_ Nanorattles@LB-Agar Substrates for In Situ Monitoring of pH

Au@Ag@mSiO_2_ nanorattles (900 μL) were mixed with 500 μL of a 5 mM ethanolic solution of 4-MBA and allowed to diffuse for 45 min. The 4-MBA-encoded nanorattles were centrifuged (4500 rpm, 5 min) twice, the first time the pellet was redispersed in 1 mL of water and the second time in 20 μL of water by sonication. For the substrate preparation, the 4-MBA-encoded nanorattles were mixed with 300 μL of molten LB-agar (10 g of tryptone, 5 g of yeast extract, 10 g of NaCl, and 15 g of agar per liter of water) containing 2% glucose and then poured in a PDMS mold (2 × 2 × 0.5 cm^3^) placed over a glass slide. Once solidified, the LB-agar substrate doped with 4-MBA-encoded Au@Ag@mSiO_2_ nanorattles was transferred to a humidity chamber to avoid dehydration. A drop of 2 μL of the bacterial suspension of *E. coli* (OD_600nm_ of 10) was spotted on the substrate and allowed to grow at 30 °C. For control experiments, substrates were also prepared in the absence of glucose.

### Bacterial Strains and Culture Conditions

Typically, bacterial cells of *P. aeruginosa* PA14 and *E. coli* MG1655 were streaked from frozen stocks onto lysogeny broth (LB) agar plates and incubated overnight at 30 °C. Single colonies of *P. aeruginosa* strains were used to inoculate 10 mL of LB medium (LB: 10 g of tryptone, 5 g of yeast extract, and 10 g of NaCl per liter of water) and grown at 37 °C with agitation (220 rpm) for 18 h. Next, the culture was washed three times with fresh LB (7000 rpm, 3 min) and the cell pellet was resuspended in 50 mL of fresh LB. The culture was incubated at 37 °C with agitation (220 rpm), and the samples were collected at various times for analysis for SERS analysis and measurement of the concentration of cells through optical density at 600 nm (OD_600nm_). *E. coli* strains were used to inoculate 10 mL of LB medium and grown at 37 °C with agitation (220 rpm) for 18 h. Next, the culture was centrifuged (4000 rpm, 10 min), and the cell pellet was resuspended in LB medium to an optical density at 600 nm (OD_600_) of 10.

### Pyocyanin Extraction Assay

Pyocyanin extraction was performed as previously reported.^34^ Aliquots of bacterial culture of *P. aeruginosa* were collected at 4, 7, and 24 h of bacterial growth and centrifuged at 4000 rpm for 10 min. The supernatant (4 mL) was filtered using a syringe filter (0.2 μm pore size) and subsequently mixed with 6.7 mL of chloroform under vigorous agitation to extract pyocyanin to the organic phase. Next, the sample was centrifuged at 9000 rpm for 6 min and 3 mL of the organic phase was collected and mixed with 1.5 mL of a 0.2 M HCl solution to extract pyocyanin to the aqueous phase. Finally, the sample was centrifuged (9000 rpm, 6 min) and the aqueous phase containing pyocyanin was analyzed by UV–vis-NIR spectroscopy at 520 nm.

### SERS Measurements

SERS experiments were conducted in a Renishaw InVia Reflex system, composed of a confocal microscope, a CCD camera, high-resolution diffraction gratings (1200 grooves cm^–1^), monochromatic light source (laser 785 nm), and optical components (filters and lenses).

### Detection and Quantification of Secreted Pyocyanin in Planktonic Cultures

First, a calibration curve was obtained. Thus, aliquots of 100 μL of Au@Ag@mSiO_2_ nanorattles in ethanol were added each to 100 μL water and centrifuged at 4500 rpm, 5 min. The pellets were resuspended in 500 μL of commercial pyocyanin solutions in LB (diluted 10× in water). The concentration of pyocyanin varied from 0.5 to 50 μM. SERS spectra of liquid samples were collected using a Renishaw macrosampler accessory, using a 785 nm laser line, 15× objective, 53.1 mW maximum power, and an acquisition time of 10 s. The detection and quantification of secreted pyocyanin were done similarly resuspending the pellets in 500 μL of bacteria-free supernatants obtained from cultures of *P. aeruginosa* at different growth times (previously diluted 10× in water and centrifuged). After 30 s of sonication, the samples were analyzed by SERS.

### SERS Measurements

SERS spectra of Au@Ag@mSiO_2_ nanorattle-doped LB-agar substrates adjusted at different pH values were performed using laser excitation of 785 nm, 10× objective, 8.22 mW maximum power, 10 s acquisition time, and 3 accumulations. Sixteen spectra were randomly recorded in an area of 16 mm^2^. SERS mapping on Au@Ag@mSiO_2_ nanorattle-doped LB-agar substrates during bacterial growth was performed using laser excitation of 785 nm, 10× objective, 8.22 mW maximum power, 1 s acquisition time, and 10 accumulations. SERS images were obtained using a point-mapping method at the selected area (1.8 mm^2^), in which each SERS spectrum is measured every 150 μm. All data were processed using WiRE software v 4.3 (Renishaw, U.K.).

## Supporting Information Available

Additional TEM images and EDX elemental mapping; additional AFM images in the collapsed and swollen states; SERS spectra and calibration curve for the detection of pyocyanin in water and in LB medium; comparison of the performance of Au@Ag@mSiO2 core-shell nanorods vs Au@Ag@mSiO2 nanorattles and additional pH-dependent SERS measurements (PDF)
