# Supplementary material for: Plasmonic Au@Ag@mSiO2 Nanorattles for In Situ Imaging of Bacterial Metabolism by Surface-Enhanced Raman Scattering Spectroscopy
Source: ACS Appl Mater Interfaces. 2021 Dec 20;13(51):61587–97. doi: 10.1021/acsami.1c21812 (PMC8719315; doi:10.1021/acsami.1c21812)
Supplement: Supplementary file 1 — am1c21812_si_001.pdf [file am1c21812_si_001.pdf]

SUPPORTING INFORMATION

for

Plasmonic Au@Ag@mSiO$_2$ Nanorattles for in situ Imaging of Bacterial Metabolism by Surface-Enhanced Raman Scattering Spectroscopy

Sarah De Marchi,[1,2] Daniel García-Lojo,[1,2] Gustavo Bodelón,[1,2,*] Jorge Pérez-Juste,[1,2,*] and Isabel Pastoriza-Santos[1,2,*]

[1] CINBIO, Universidade de Vigo, Departamento de Química Física, Campus Universitario As Lagoas, Marcosende, 36310 Vigo, Spain

[2] Galicia Sur Health Research Institute (IIS Galicia Sur), SERGAS-UVIGO, 36310 Vigo, Spain

Email: gbodelon@uvigo.es
juste@uvigo.es
pastoriza@uvigo.es

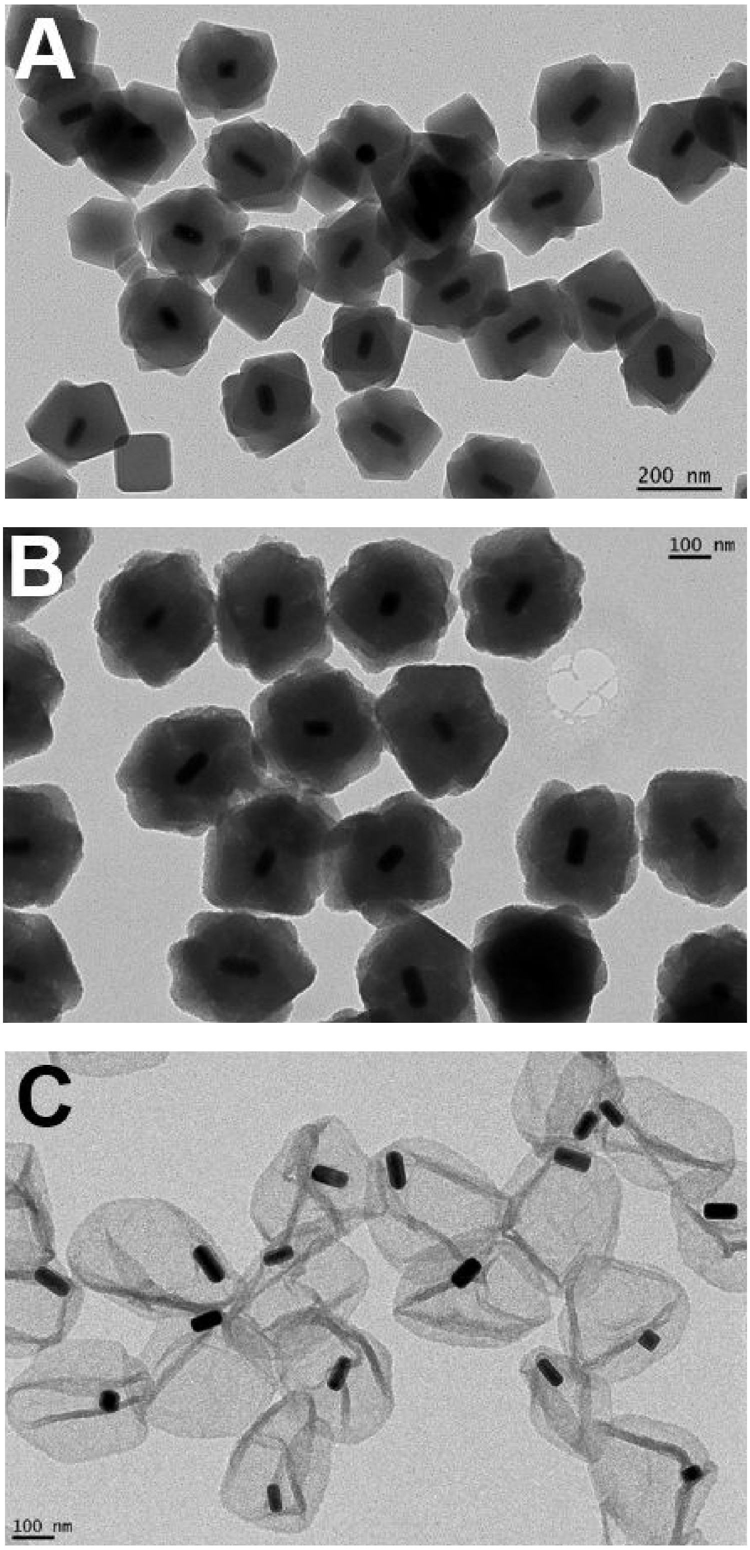

Figure S1. Representative TEM images of **(A)** Au@Ag@ZIF-8, **(B)** Au@Ag@ZIF-8@mSiO_2, and **(C)** Au@Ag@mSiO_2 nanorattles.

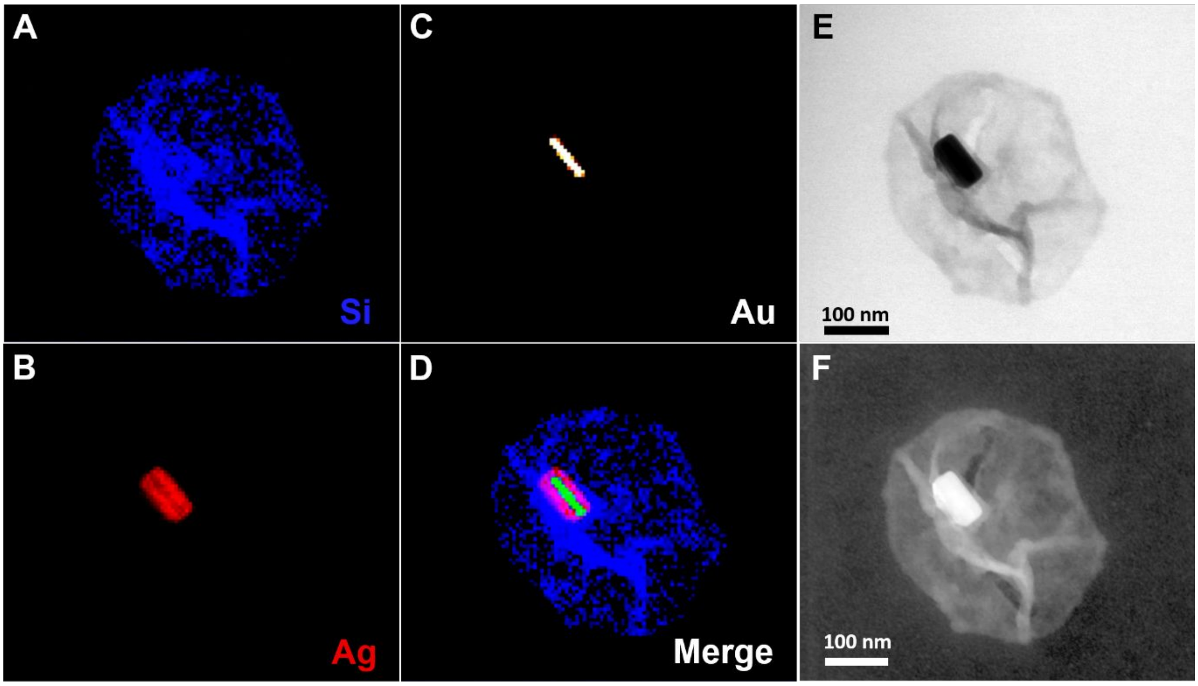

Figure S2. **(A-D)** EDX elemental mapping of an Au@Ag@msiO_2 nanorattle showing the Si in blue, Ag in red and Au in yellow. **(E-F)** Corresponding bright field (E) and dark field (F) transmission electron microscopy (TEM) images.

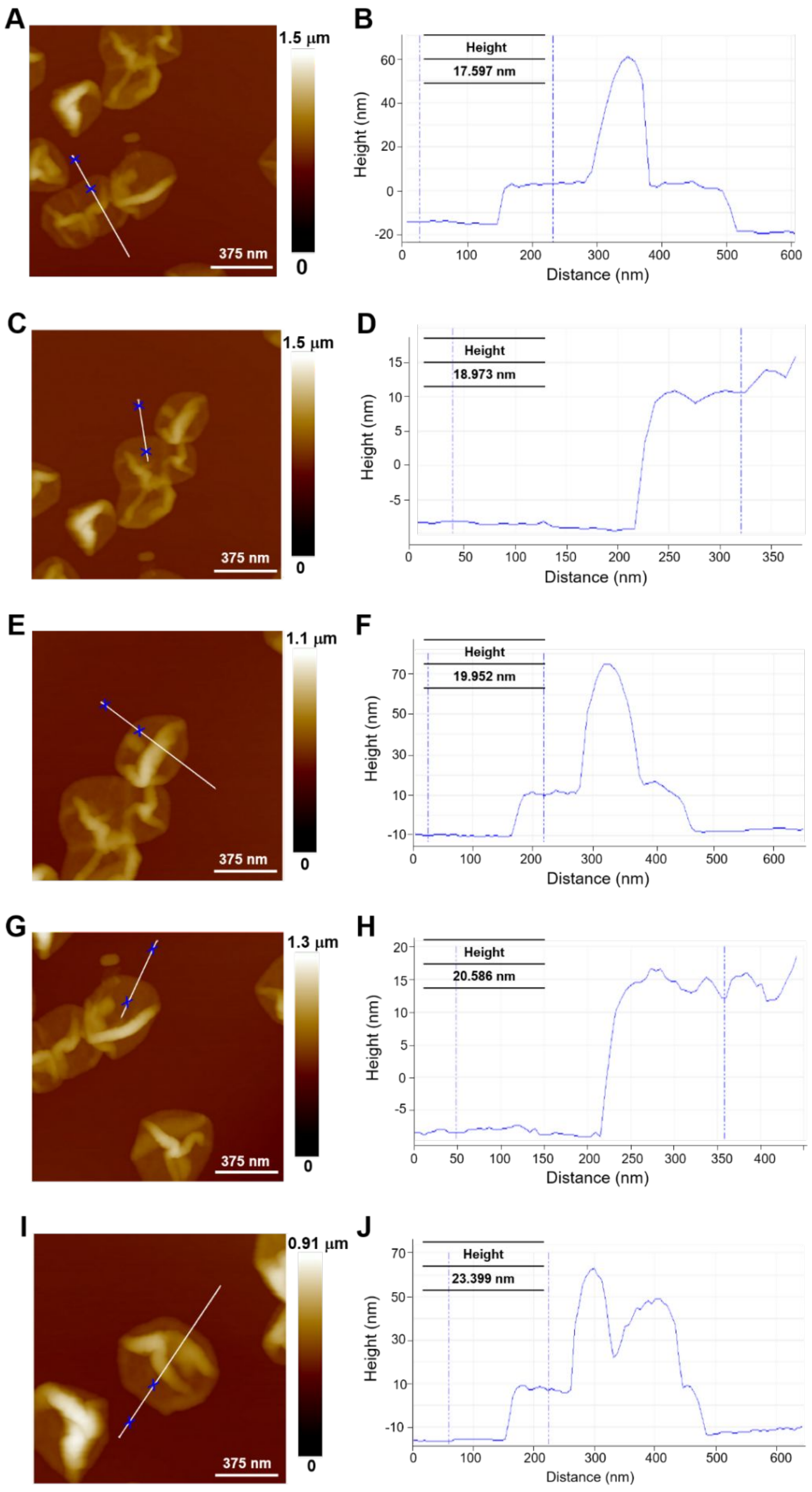

Figure S3. AFM topographic height images **(A, C, E, G, I)** and height profiles **(B, D, F, H, J)** of dried Au@Ag@mSiO_2 nanorattles.

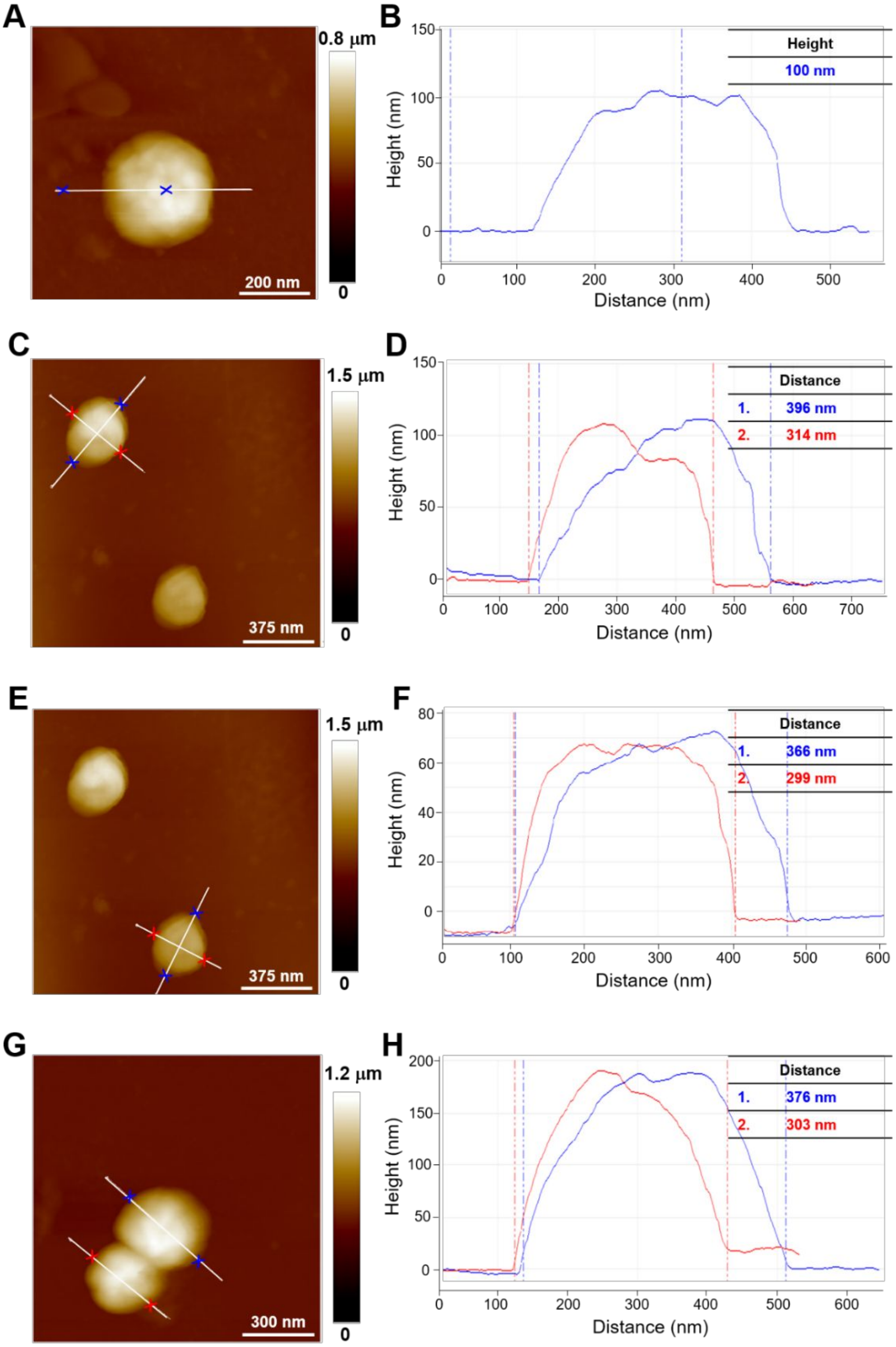

Figure S4. AFM topographic height images **(A, C, E, G)** and height profiles **(B, D, F, H)** of hydrated Au@Ag@mSiO_2 nanorattles.

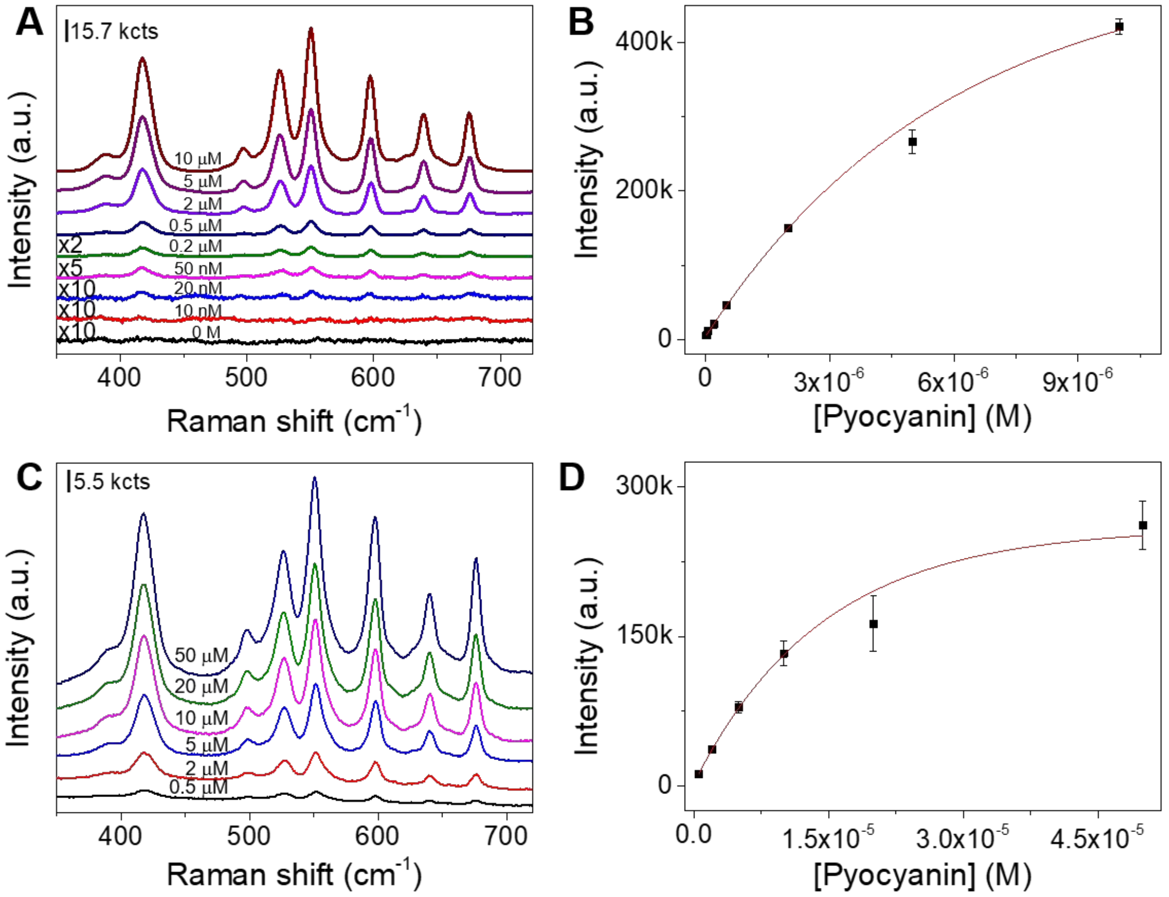

Figure S5. (A) SERS spectra of commercial pyocyanin in water at different concentrations recorded using the plasmonic $mSiO_2$ nanorattles substrates. The peaks observed between 400-600 cm^{-1} correspond to different ring deformations from pyocyanin. **(B)** SERS intensity at 675 cm^{-1} as a function of pyocyanin concentration. The red line is the Langmuir isotherm fit. Error bars indicate the standard deviation of three measurements. **(C)** SERS spectra of commercial pyocyanin in LB medium at different concentrations recorded on $Au@Ag@SiO_2$ nanorattles. **(D)** SERS intensity at 675 cm^{-1} as a function of pyocyanin concentration. The red line is the Langmuir isotherm fit. Error bars indicate the standard deviation of three different measurements. SERS analysis was carried out with an excitation laser line at 785 nm employing a 15x objective, maximum power of 53.1 mW, and an acquisition time of 10s.

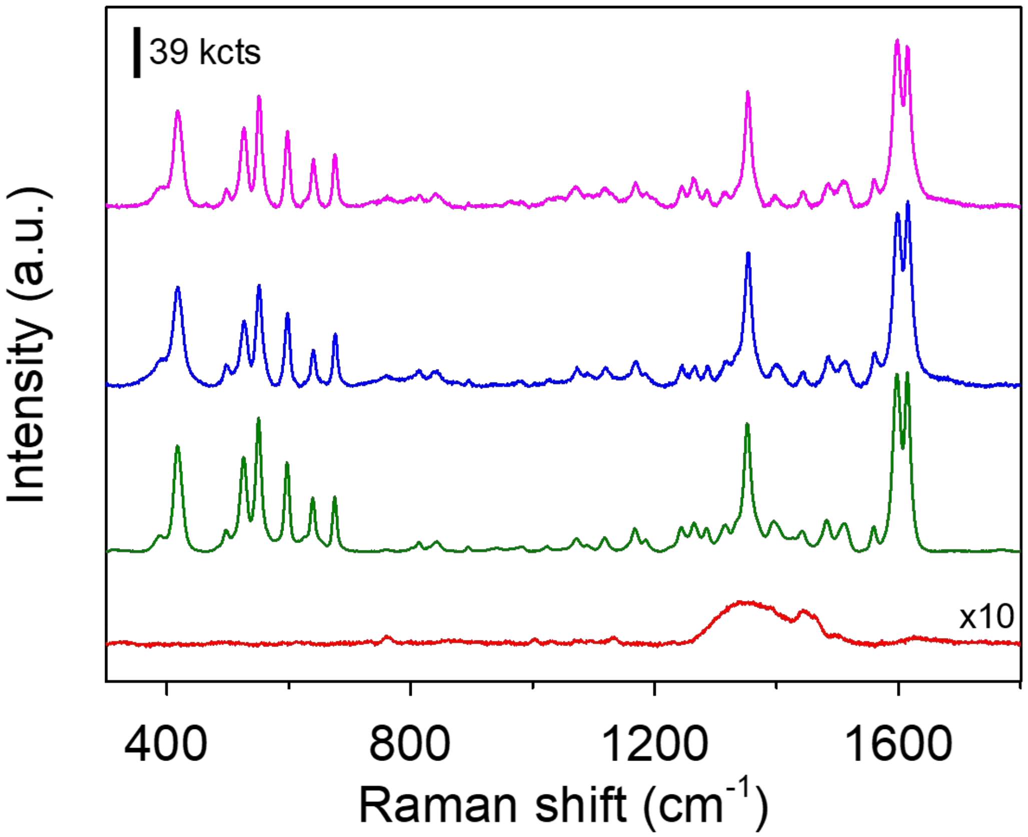

Figure S6. SERS spectra recorded in a colloidal dispersion of Au@Ag@mSiO_2 nanorattles in: LB medium (red line), pure water containing commercial pyocyanin (green line), LB medium containing commercial pyocyanin (blue line), and secreted pyocyanin medium after 8 h of bacterial growth (pink line). SERS measurements were carried out at 785 nm with a 10x objective, 8.22 mW of maximum power, 1 accumulation, and an acquisition time of 10 s.

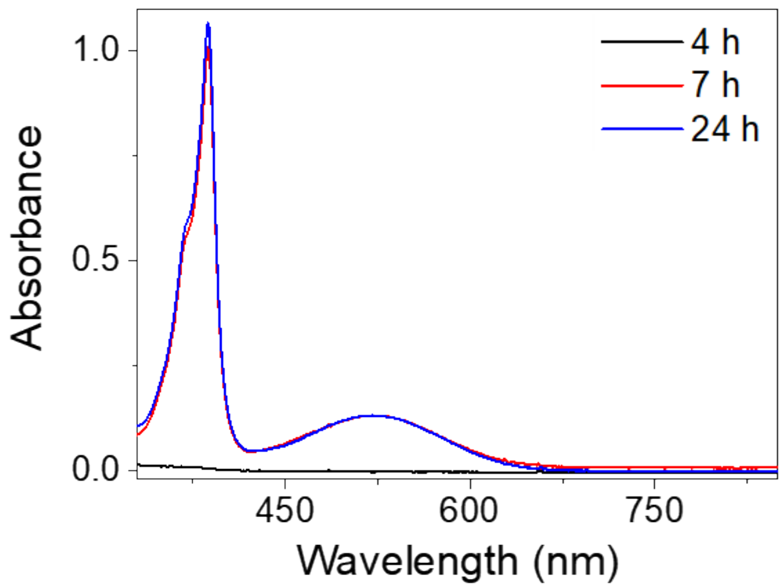

Figure S7. Absorption spectra of extracted pyocyanin at 4 h (black line), 7 h (red line) and 24 h (blue line) of bacterial growth.

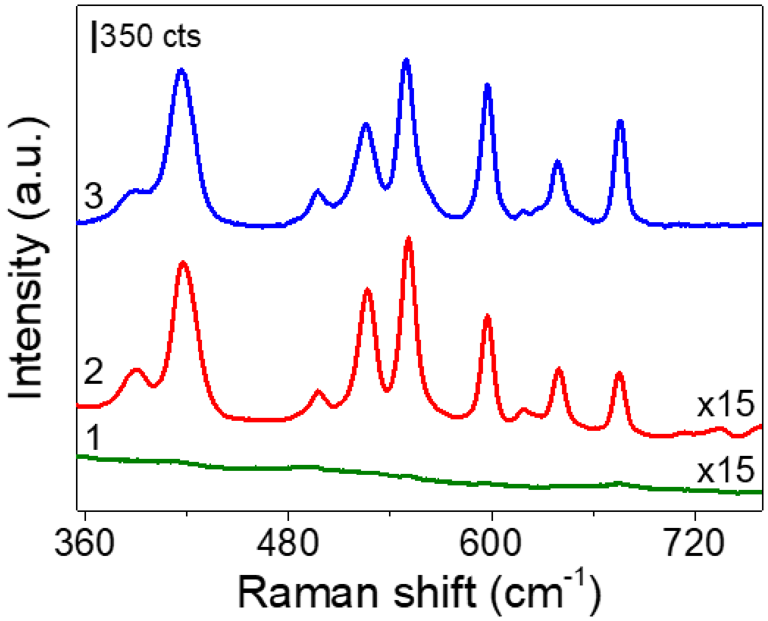

Figure S8. Comparison of the SERS signal of pyocyanin secreted by a 6h culture of *P. aeruginosa* bacteria recorded using different plasmonic substrates: (1) CTAC-stabilized Au@Ag nanorods and (2) Au@Ag@mSiO_2 nanorattles. SERS spectrum of commercial pyocyanin recorded on Au@Ag@mSiO_2 nanorattles (3).

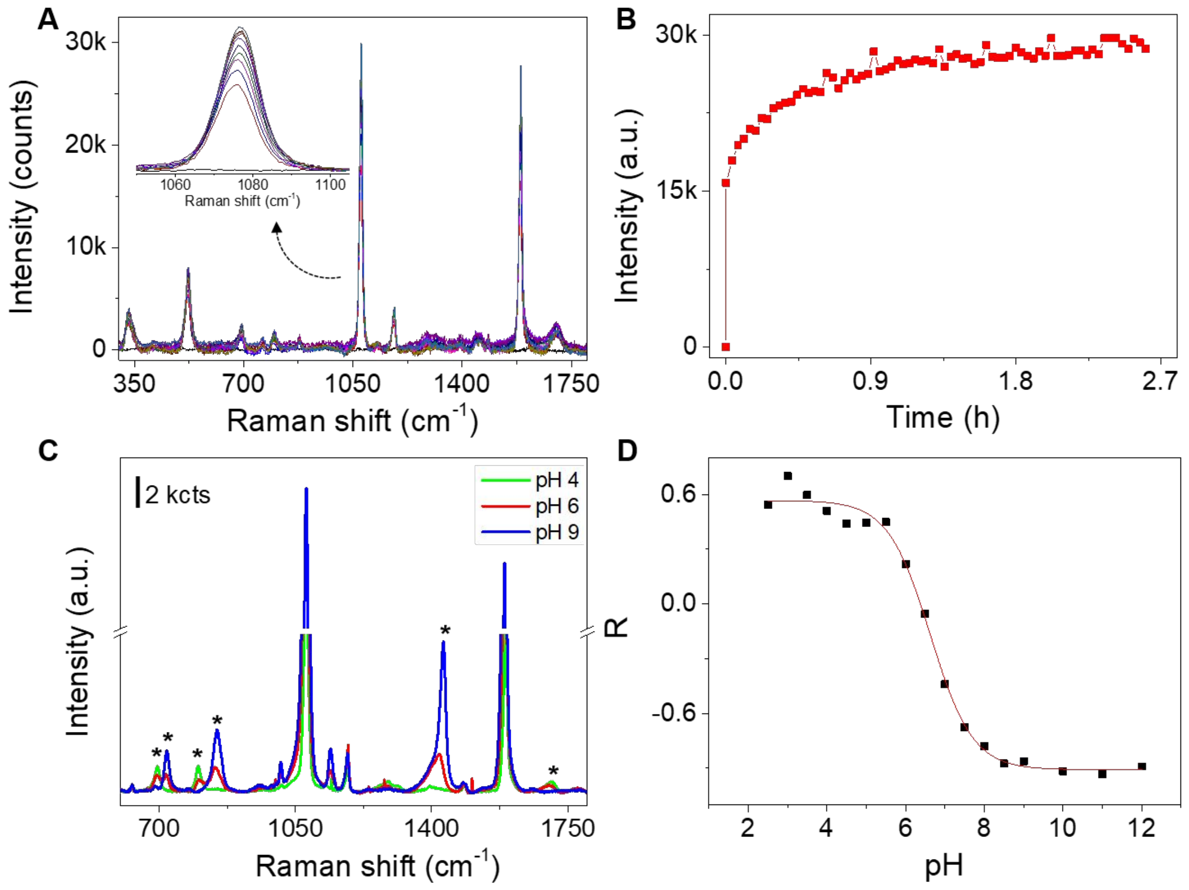

Figure S9. (A) SERS spectra of 4-MBA recorded at different times. **(B)** Time evolution of the 1076 cm^{-1} band during functionalization of plasmonic $mSiO_2$ nanorattles. **(C)** Typical SERS spectra of 4-MBA recorded in phosphate buffer at different pH´s. The asterisks indicate the pH-sensitive bands. **(D)** Experimental calibration curve of ratio $R = (A_{695} – A_{848})/(A_{695} + A_{848})$ as a function of pH (error bars show the standard deviation from the mean of n = 15 measurements). SERS measurements were carried out at 785 nm with a 10x objective, 8.22 mW of maximum power, 3 accumulations, and an acquisition time of 10 s.

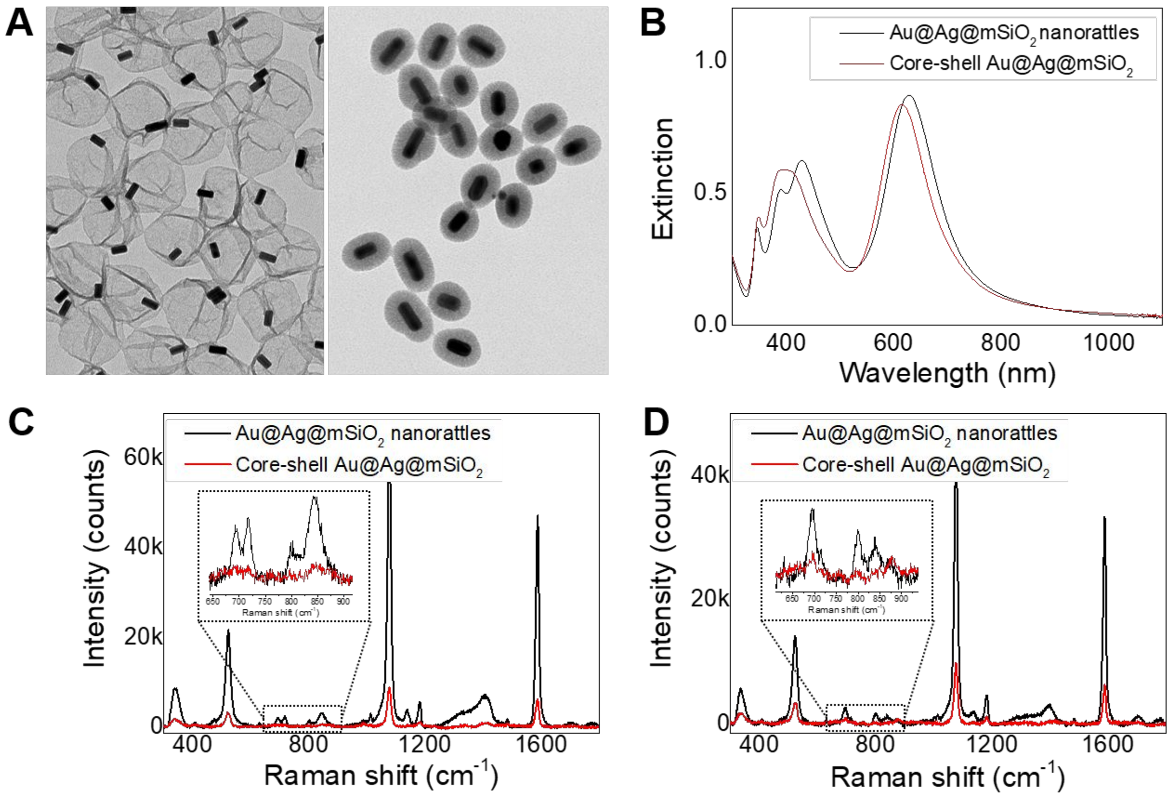

Figure S10. **(A)** Representative TEM images of hollow Au@Ag@mSiO2 nanorattles (left) and core-shell Au@Ag@mSiO2 nanoparticles (right). **(B)** UV-vis spectra of hollow Au@Ag@mSiO2 nanorattles (black line) and core-shell Au@Ag@mSiO2 nanoparticles (red line). **(C-D)** SERS spectra of Au@Ag@mSiO2 nanorattles (black line) and core-shell Au@Ag@mSiO2 nanoparticles (red line) in buffered LB medium at pH 6.8 (C) and buffered LB medium at pH 5.7 (D). Insets denote pH-sensitive bands.

Comparison of the performance of Au@Ag@mSiO_2 core-shell nanorods vs Au@Ag@mSiO_2 nanorattles. 4-MBA codified Au@Ag@mSiO_2 nanorattles (Figure 10A, left) and core-shell Au@Ag@mSiO_2 nanoparticles (Figure 10A, right) with similar optical properties (Figure 10B) were synthesized (see the experimental section for details) and their SERS activities were studied. The particles were encoded with 4-MBA in ethanol, washed with water, and resuspended first in LB medium at pH 6.8 (Figure 10C) and then at pH 5.7 (Figure 10D). Spectra of 4-MBA acquired using the Au@Ag@mSiO_2 nanorattles as substrates displayed much higher SERS signals than those obtained with core-shell Au@Ag@mSiO_2 nanoparticles in both pH´s, especially in the regions of the pH-sensitive bands. Based on these results, using ZIF-8 as a template to create a cavity between the plasmonic nanoparticle and the mSiO_2 shell was a key factor to obtain a more sensitive sensor.

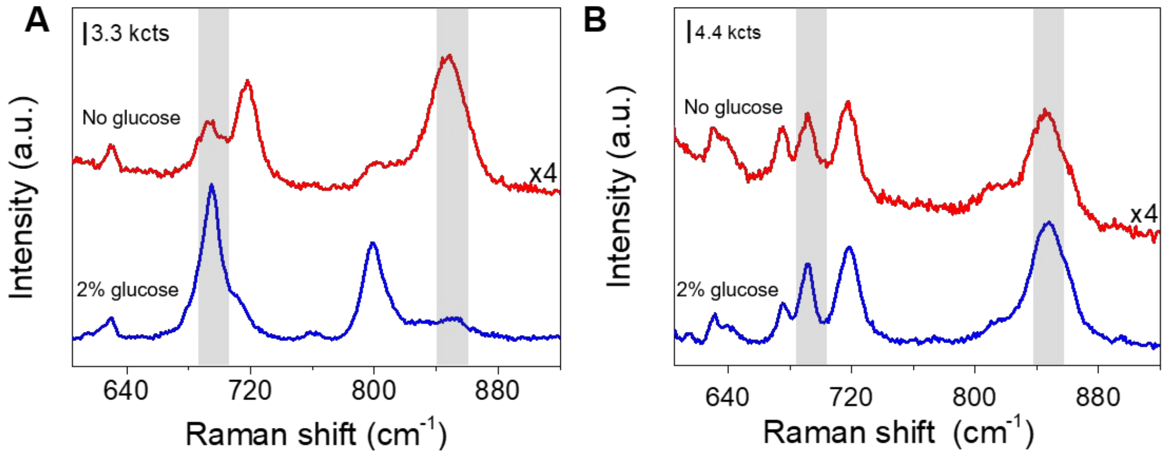

Figure S11. SERS spectra of 4-MBA-encoded $mSiO_2$ nanorattles in bacterial cultures of **(A)** *E. coli* and **(B)** *P. aeruginosa* supplemented with 2% of glucose (blue line) and without glucose (red line). The bands at 695 and 848 cm^{-1} are indicated with grey bars. SERS measurements were carried out at 785 nm with a 50x objective, 5.82 mW of maximum power, and an acquisition time of 10 s.

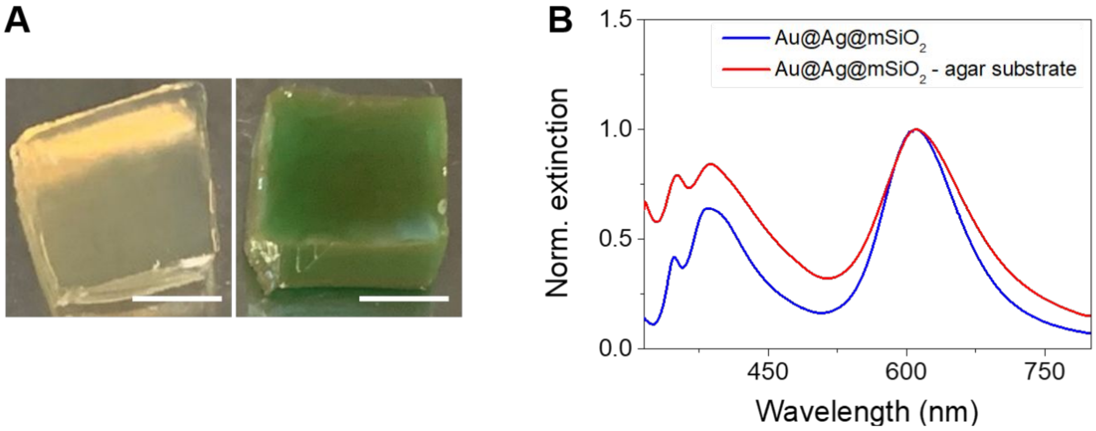

Figure S12. (A) Optical images of LB-agar (left) and 4-MBA-encoded plasmonic nanorattles embedded in LB-agar (right). Scale bars represent 0.5 cm. **(B)** UV-vis extinction spectra of Au@Ag@mSiO_2 nanorattles (blue line) and nanorattles@LB-agar substrates (red line).

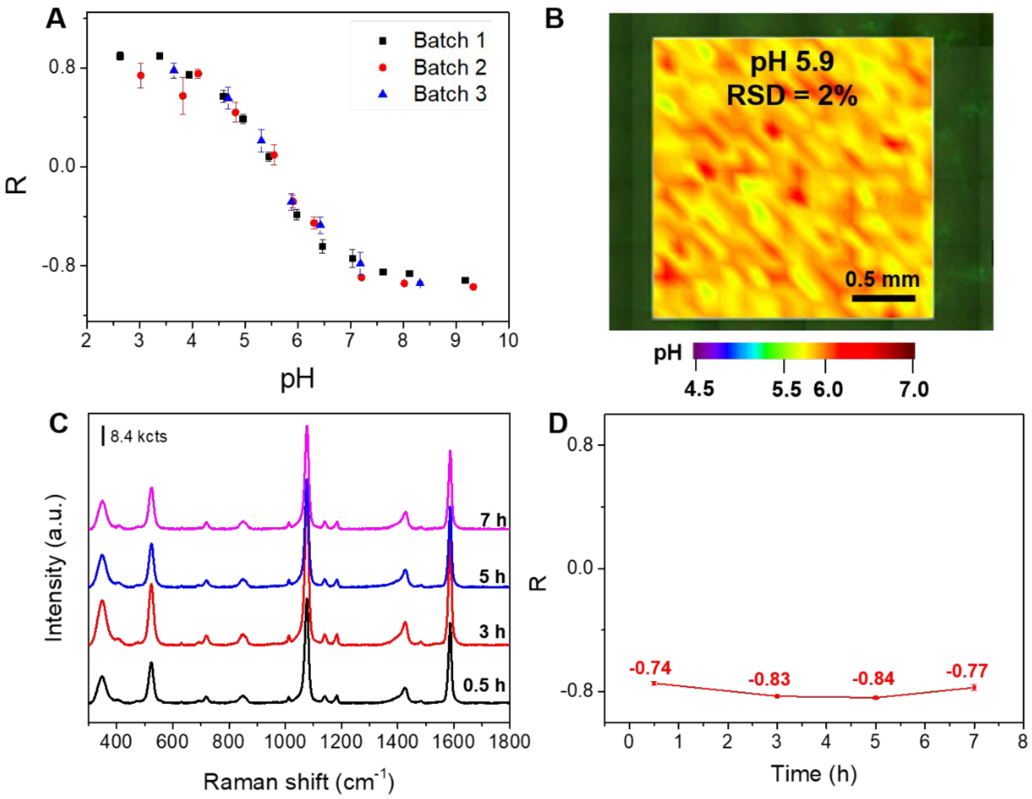

Figure S13. (A) Experimental calibration curves of ratio R = (A_{695} – A_{848})/(A_{695} + A_{848}) as a function of pH. The error bars show the standard deviation from the mean of n = 16 measurements for each batch of plasmonic nanorattles. SERS measurements were carried out at 785 nm with a 10x objective, 8.22 mW of maximum power, 3 accumulations, and an acquisition time of 10 s. **(B)** pH distribution map of a selected area of an Au@Ag@mSiO_2 nanorattles doped LB-agar substrate with the pH adjusted at 5.9. SERS measurements were carried out at 785 nm with a 10x objective, 56.5 mW of maximum power, 3 accumulations, and an acquisition time of 0.5 s. **(C)** SERS spectra recorded in Au@Ag@mSiO_2 nanorattles doped LB-agar substrate without glucose at 0.5, 3, 5 and 7 h of bacterial growth. **(D)** Calculated ratio R = (A_{695} – A_{848})/(A_{695} + A_{848}) as a function of bacterial growth time.

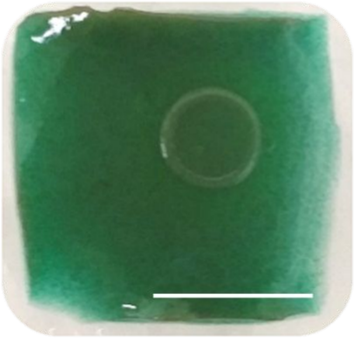

Figure S14. Optical image of an *E. coli* colony grown on the nanorattles@LB-agar substrate. Scale bar represents 1 cm.

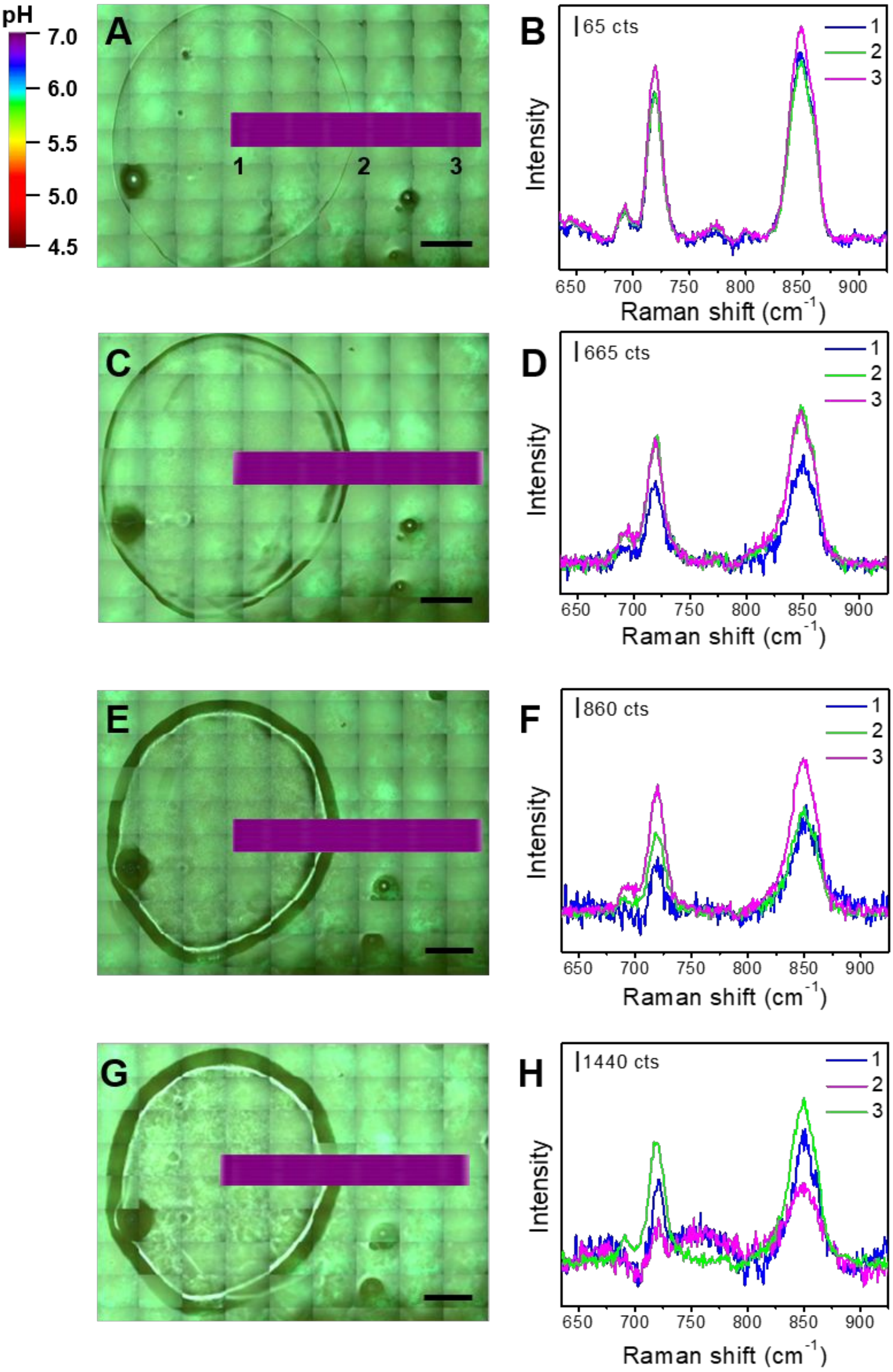

Figure S15. (A, C, E, G) SERS maps showing the values of the calculated relative ratio R (eq. 1) in a colony of *E. coli* grown on a nanorattles@LB-agar substrate without glucose and **(B, D, F, H)** SERS spectra from points 1, 2, and 3 of these maps. Scale bars represent 1 mm. SERS measurements were carried out at 785 nm with a 10x objective, 8.22 mW of maximum power, 10 accumulations, and an acquisition time of 1 s.

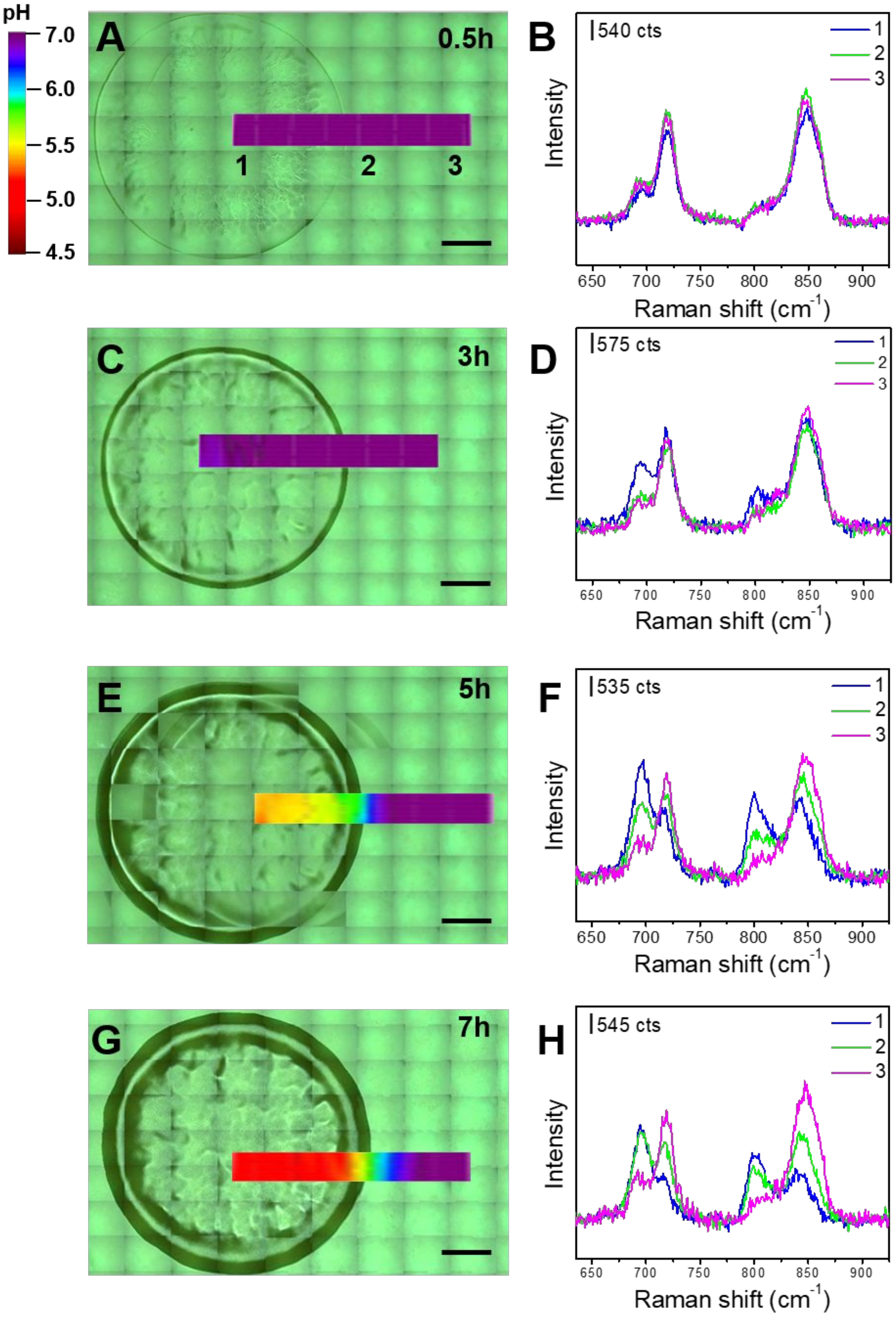

Figure S16. (A, C, E, G) SERS maps showing the values of the calculated relative ratio R (eq. 1) in a colony of *E. coli* grown on a nanorattles@LB-agar substrate supplemented with glucose and **(B, D, F, H)** SERS spectra from points 1, 2, and 3 of these maps. Scale bars represent 1 mm. SERS measurements were carried out at 785 nm with a 10x objective, 8.22 mW of maximum power, 10 accumulations, and an acquisition time of 1 s.
